# Interpretable Graph–Temporal–Spectral Fusion for Precursor-Related Anomaly Detection in Underground Mine Sensor Networks

**DOI:** 10.3390/s26165305

**Published:** 2026-08-21

**Authors:** Shuren Mao, Yunpei Liang, Quangui Li

**Affiliations:** 1 State Key Laboratory of Coal Mine Disaster Dynamics and Control, Chongqing University, Chongqing 400044, China; mshuren@126.com (S.M.); liqg@cqu.edu.cn (Q.L.); 2 School of Resources and Safety Engineering, Chongqing University, Chongqing 400044, China

**Keywords:** underground mine sensor networks, multi-source sensor fusion, precursor-related anomaly detection, graph neural networks, temporal–spectral modeling, interpretable monitoring

## Abstract

**Highlights:**

An engineering-prior sensor graph is integrated with temporal–spectral modeling for underground mine anomaly detection. GasNet combines GCN, TimesNet, and cross-attention to model heterogeneous multi-source sensor dependencies. Field evaluation achieved full coverage of the annotated precursor-related abnormal intervals with a low false-alarm rate. Multi-level interpretation links feature-fusion behavior with sensor-time warning evidence.

**What are the main findings?**

- GasNet integrates an engineering-prior sensor graph, GCN, TimesNet, and cross-attention to detect precursor-related abnormal intervals from multi-source underground mine sensor data.
- Field validation on the 31002 working face showed that GasNet achieved high detection sensitivity, with a Recall of 1.000, an F1-score of 0.937, and zero missed detections.

**What are the implications of the main findings?**

- Engineering-prior sensor graph construction can improve abnormal-state detection by incorporating ventilation pathways, sensor deployment, and equipment-coupling relationships.
- The proposed multi-level interpretability framework provides traceable sensor-time evidence for on-site warning review and engineering verification.

**Abstract:**

Underground coal mine safety monitoring relies on multi-source sensor networks, but abnormal-state detection remains challenging because methane, airflow, dust, and equipment-operation signals are non-stationary, heterogeneous, and constrained by ventilation and mining disturbances. This study proposes GasNet, an interpretable engineering-prior graph–temporal–spectral framework for precursor-related anomaly detection. Variables are organized into methane-related core sensors and environmental–operational modulation sensors. A directed sensor graph is constructed using ventilation causality, sensor deployment, and shearer-coupling relationships. GasNet then integrates a graph convolutional network for spatial–topological modeling, TimesNet for temporal–spectral pattern extraction, and cross-attention for adaptive feature fusion. An unsupervised reconstruction strategy identifies intervals deviating from learned normal production patterns. Field validation was conducted on the 31002 fully mechanized working face of the Xinyuan Coal Mine, where eight precursor-related abnormal intervals were annotated from monitoring data and field records. GasNet achieved a Precision of 0.881, a Recall of 1.000, an F1-score of 0.937, a false-alarm rate of 0.0017, and zero missed detections, with the highest F1-score among seven time-series baselines. Interpretability analysis further provided feature-fusion and sensor-time evidence for warning review. These results support the feasibility of GasNet for interpretable anomaly detection in the investigated working face.

## 1. Introduction

Underground coal mine safety monitoring increasingly relies on multi-source sensor networks to perceive abnormal operating states in complex production environments. Multi-source monitoring systems continuously record methane concentration, airflow, dust, and equipment-operation signals, providing a data basis for abnormal-state detection and early warning in underground working faces [1,2,3,4,5]. However, unlike ordinary industrial time series, underground mine monitoring signals are usually non-stationary, heterogeneous, and strongly coupled. Their variations are affected not only by the intrinsic evolution of gas emission, but also by ventilation pathways, sensor deployment, shearer operation, geological disturbance, and short-term production activities [6,7,8]. Therefore, reliable abnormal-state identification requires a model that can jointly represent temporal evolution, sensor-topological dependence, and engineering context.

Coal and gas outburst is one of the most serious dynamic hazards in underground coal mining. It is associated with the coupled effects of in situ stress, gas pressure, coal structure, geological disturbance, and mining activity [9,10,11]. In practice, direct accident samples are extremely rare because outburst-prevention measures are implemented before severe accidents occur. As a result, it is usually difficult to construct a large supervised dataset containing sufficient real outburst cases. Previous studies have attempted to address this problem through machine learning, Bayesian modeling, small-sample transfer learning, and optimized neural-network methods [12,13,14]. More realistically, precursor-related evidence in coal mine production is obtained from both continuous sensor monitoring and field-operation records. Precursor-related evidence is available from continuous sensor anomalies and contemporaneous field-operation records, including abnormal gas responses, drilling phenomena, geological disturbances, and intervention measures [15,16,17]. These heterogeneous data sources do not represent deterministic outburst accidents, but they provide important evidence for identifying abnormal monitoring intervals that require engineering attention. Accordingly, the objective of this study is not to predict the occurrence of an actual outburst accident, but to detect precursor-related abnormal intervals from multi-source underground mine sensor data.

Existing studies on mine gas monitoring have widely used data-driven models for methane concentration prediction and early warning. Recurrent neural networks, encoder–decoder structures, Transformer-based models, attention-based spatio-temporal models, graph convolutional encoder–decoder models, and expert-knowledge-guided graph models have improved the ability to learn nonlinear temporal and spatial patterns from monitoring data [18,19,20,21,22]. Multi-information fusion has also been applied to the monitoring and early warning of other mining dynamic hazards under uncertainty, indicating the value of integrating heterogeneous monitoring sources for safety decision support [23]. However, most existing approaches still have three limitations when applied to underground mine sensor networks. First, many models mainly focus on temporal dependency extraction and treat different monitoring variables as independent or homogeneous input channels. This may cause the model to confuse normal production-induced fluctuations with precursor-related abnormal deviations. Second, the physical relationships among sensors are often insufficiently exploited. In a working face, gas-related signals are constrained by ventilation direction, roadway layout, sensor position, and equipment-induced disturbance. These dependencies are directional and physically meaningful rather than arbitrary statistical correlations. Third, many deep learning models provide limited interpretability. For underground safety monitoring, a binary warning or an anomaly score is insufficient; engineers also need to know which sensor relationships, monitoring variables, and temporal segments support the warning output. This requirement is consistent with the broader concern that explainable artificial intelligence is essential for improving the transparency and trustworthiness of data-driven decision systems [24,25]. Existing graph-based studies in mine monitoring have mainly focused on methane-concentration forecasting, spatio-temporal dependency learning, and expert-knowledge-assisted risk representation. However, the explicit construction of a directed sensor graph from ventilation pathways, actual sensor deployment, and equipment-coupling mechanisms for unsupervised precursor-related anomaly detection remains insufficiently explored.

To address these gaps, this study proposes GasNet, an interpretable graph–temporal–spectral framework for precursor-related anomaly detection in underground mine sensor networks. The key idea is to explicitly encode ventilation pathways, sensor deployment, and shearer-related coupling into a directed engineering-prior graph and jointly model this spatial topology with temporal–spectral evolution. GasNet combines graph-based sensor dependency modeling, TimesNet-based temporal–spectral representation, cross-attention fusion, and unsupervised reconstruction to identify deviations from normal production patterns. A multi-level interpretation scheme is further introduced to provide feature-fusion and sensor-time evidence for warning review. Therefore, the methodological distinction lies in how domain-specific engineering relationships are formalized as a directed sensor topology and integrated with temporal–spectral anomaly modeling, rather than in introducing a new graph neural-network operator itself.

The main contributions of this study are summarized as follows:

1. An engineering-prior directed sensor graph is constructed from ventilation pathways, sensor deployment, and equipment-operation relationships, rather than being learned solely from statistical correlations, enabling physically constrained spatial information aggregation.
2. A graph–temporal–spectral fusion framework combining GCN, TimesNet, and cross-attention is developed for reconstruction-based precursor-related anomaly detection under unsupervised training.
3. A multi-level interpretation framework combines feature-fusion analysis and distilled sensor-time visualization to support engineering review of abnormal warnings.

The remainder of this paper is organized as follows. Section 2 presents the proposed GasNet framework, including the sensor-role hierarchy, engineering-prior graph construction, graph–temporal–spectral fusion architecture, reconstruction-based anomaly-detection strategy, and interpretability module. Section 3 describes the field case, sensor deployment, data preprocessing, abnormal interval annotation, experimental settings, baseline comparison, ablation study, and sensitivity analysis. Section 4 presents the interpretability analysis and on-site warning tracing results. Section 5 discusses the engineering implications, limitations, and future work. Section 6 concludes this study.

## 2. Proposed GasNet Framework

This section presents the proposed GasNet framework for precursor-related anomaly detection using multi-source underground mine sensor data. The framework is designed according to two considerations. First, monitoring variables collected from an underground working face have different sensing roles. Methane sensors directly reflect gas emission, accumulation, and migration, whereas airflow, dust, and shearer-operation sensors provide environmental and operational context that modulates methane responses. Therefore, these variables should not be treated as homogeneous input channels. Second, the underground sensor network is not an unordered set of time series. Sensor responses are constrained by ventilation pathways, roadway layout, sensor deployment, and equipment-induced disturbance. These engineering relationships provide useful prior knowledge for graph-based sensor fusion.

Accordingly, GasNet consists of five components: a multi-source sensor-role hierarchy, an engineering-prior sensor graph, a graph–temporal–spectral fusion architecture, a reconstruction-based anomaly-detection strategy, and a multi-level interpretability module. The sensor-role hierarchy defines how different monitoring variables contribute to abnormal-state perception. The engineering-prior graph encodes spatial and operational relationships among sensors. The GasNet architecture then integrates GCN-based spatial–topological modeling, TimesNet-based temporal–spectral modeling, and cross-attention-based feature fusion. Finally, reconstruction errors are used to detect monitoring intervals that deviate from normal production patterns, and the interpretability module provides traceable evidence for on-site warning review.

### 2.1. Multi-Source Sensor Variables and Hierarchical Monitoring Roles

Dynamic abnormal-state monitoring in underground coal mines depends on the coordinated responses of multiple sensor types. Methane concentration is the most direct monitoring signal for gas emission and accumulation, but its observed value is affected by airflow dilution, coal fragmentation, shearer operation, and sensor location. Therefore, a methane fluctuation cannot be interpreted independently from its environmental and operational context. To support physically meaningful sensor fusion, the monitoring variables are organized into two hierarchical roles: methane-related core sensors and environmental–operational modulation sensors, as shown in Table 1.

Let the multivariate monitoring sequence be denoted as $X\;\in {\mathbb{R}}^{T\;\times N}$, where T is the input time window length and N is the number of monitoring variables. The sensor vector at time step t is defined as

(1) $$x_{t}=[x_{t}^{core},x_{t}^{mod}],$$

where $x_{t}^{core}$ denotes methane-related core sensor variables and $x_{t}^{mod}$ denotes environmental and operational modulation variables.

After defining the multivariate monitoring sequence, the selected sensor variables are further interpreted according to their physical sensing roles and their functional contributions to GasNet. Specifically, the methane-related core variables are regarded as direct gas-response sensors, while the modulation variables are further divided into environmental modulation sensors and operational modulation sensors. The three sensor roles are described as follows.

1. Methane-related core sensors. Methane concentration sensors are treated as core response sensors because they directly characterize gas emission, accumulation, and migration in the working face. Sensors installed at different locations capture different aspects of the gas-response process. For example, upper-corner methane reflects local gas accumulation near the goaf-side region, working-face methane captures gas emission during cutting and face advancement, return-airway methane represents the integrated gas outflow of the working face, and intake-airflow methane provides upstream background information. In this study, the methane-related core sensors include sensors installed at the upper corner, working face, return airway, intake airflow, and onboard shearer position.
2. Environmental modulation sensors. Airflow and dust sensors provide environmental context for interpreting methane responses. Wind velocity affects methane concentration through ventilation dilution. A decrease in methane concentration caused by increased airflow does not necessarily indicate reduced gas emission, whereas stable methane concentration under weakened airflow may imply an abnormal emission state. Dust concentration reflects coal fragmentation intensity and may increase simultaneously with gas-related abnormal responses under strong mining disturbance. Therefore, airflow and dust sensors are used as contextual variables rather than direct warning variables.
3. Operational modulation sensors. Shearer-operation sensors describe the production disturbance imposed on the coal body. Traction speed, cutting current, frequency-conversion output current, and shearer position reflect mining intensity, cutting load, and the spatial location of the disturbance source. These operational variables can cause normal fluctuations in methane and dust signals during routine production. By incorporating them as modulation variables, GasNet can learn whether methane variations are consistent with normal production disturbance or deviate from the learned normal coupling pattern.

Through this hierarchical organization, GasNet learns the coupling relationship between methane-related core responses and environmental–operational modulation variables. The purpose is not to trigger warnings from any single sensor fluctuation, but to identify monitoring intervals in which the multi-source sensor relationship significantly deviates from normal production behavior.

### 2.2. Engineering-Prior Sensor Graph Construction

A critical limitation of standard deep learning models in underground mine monitoring is that they usually treat sensor variables as independent channels or fully connected inputs, thereby ignoring the physical organization of the sensor network. In an underground working face, methane migration and sensor responses are constrained by ventilation pathways, roadway layout, sensor deployment, and equipment-induced disturbance. To incorporate these engineering relationships into the model, a sensor graph is constructed as G=(V,E,A), where *V* denotes the set of sensor nodes, *E* denotes the set of edges, and $A\in {\mathbb{R}}^{N\times N}$ denotes the adjacency matrix. The graph is represented by a binary directed adjacency matrix, *A*, where $A_{\mathrm{ij}}=1$ indicates a directed edge from sensor *i* to sensor *j* and $A_{ij}=0$ otherwise. Self-loops are retained for all nodes.

Unlike purely data-driven graph learning methods, which may generate physically uninterpretable connections, the proposed graph is mainly constructed from engineering priors. Statistical correlation is used only as supplementary evidence for reinforcing physically plausible sensor relationships. The graph construction follows three rules.

1. Ventilation causality. Methane and dust signals are transported and diluted along the airflow direction. Therefore, directed edges are established from upstream sensors to downstream sensors according to the ventilation route and sensor deployment. In the studied working face, methane sensors located on the intake side, upper corner, working face, and return-airway side were connected according to their relative positions along the airflow path. This rule enables the graph to represent spatial propagation relationships among gas-related sensor responses. Directed edges are therefore established according to the relative sensor positions and the principal airflow direction, allowing information to propagate along physically meaningful ventilation pathways.
2. Equipment coupling. The shearer is the main moving disturbance source during mining. Its operating variables, such as traction speed, cutting current, frequency-conversion output current, and position, affect methane and dust responses through coal cutting, coal fragmentation, and local disturbance of gas emission. For equipment-operation relationships, variables describing the same equipment or production process are connected according to their functional coupling. Strongly coupled operating variables may be connected bidirectionally, while equipment-induced disturbances are transmitted to environmental or methane-related sensing variables through physically meaningful intermediate sensing nodes rather than by indiscriminately connecting each operating variable to all methane sensors.
3. Correlation-based supplementary reinforcement. Although the main graph structure is determined by engineering priors, some sensor pairs may also show stable statistical coupling during normal production. To avoid introducing arbitrary correlations, the Pearson correlation coefficient (PCC) is used only to reinforce sensor pairs that are physically plausible. For two sensor series $x_{i}$ and $x_{j}$, the PCC is calculated as
  (2) $$r_{ij}=\frac{\sum_{t=1}^{T}\left(x_{i,t}-{\bar{x}}_{i})(x_{j,t}-{\bar{x}}_{j}\right)}{\sqrt{\sum_{t=1}^{T}{\left(x_{i,t}-{\bar{x}}_{i}\right)}^{2}}\sqrt{\sum_{t=1}^{T}{\left(x_{j,t}-{\bar{x}}_{j}\right)}^{2}}},$$

Pearson correlation analysis is used as supplementary evidence to examine whether candidate engineering relationships are consistent with the observed monitoring data. Correlation strength is not used as an independent automatic edge-generation criterion. Instead, it is interpreted jointly with sensor location, ventilation direction, equipment function, and the physical plausibility of the corresponding interaction. Therefore, no universal PCC threshold is used to generate the final graph.

(3) $$A_{ij}^{\mathrm{corr}}=\left\{\begin{array}{l} 1,\;\;if\;an\;engineering\;relationship\;from\;node\;i\;to\;node\;j\;is\;specified,\\ 0,\;\;otherwise. \end{array}\right.,$$

Accordingly, the proposed topology is an explicitly specified engineering-prior graph rather than a graph automatically inferred from the correlation matrix. The graph-processing sequence is as follows: the directed binary engineering-prior adjacency matrix is first constructed from the specified engineering relationships; self-loops are then added before graph convolution; finally, the resulting adjacency matrix is normalized and used for GCN message passing. The detailed normalization formulation is given in Section 2.3.1. The specific graph instantiated for the field case is described in Section 3.3.

### 2.3. GasNet Architecture

**Figure 1:**
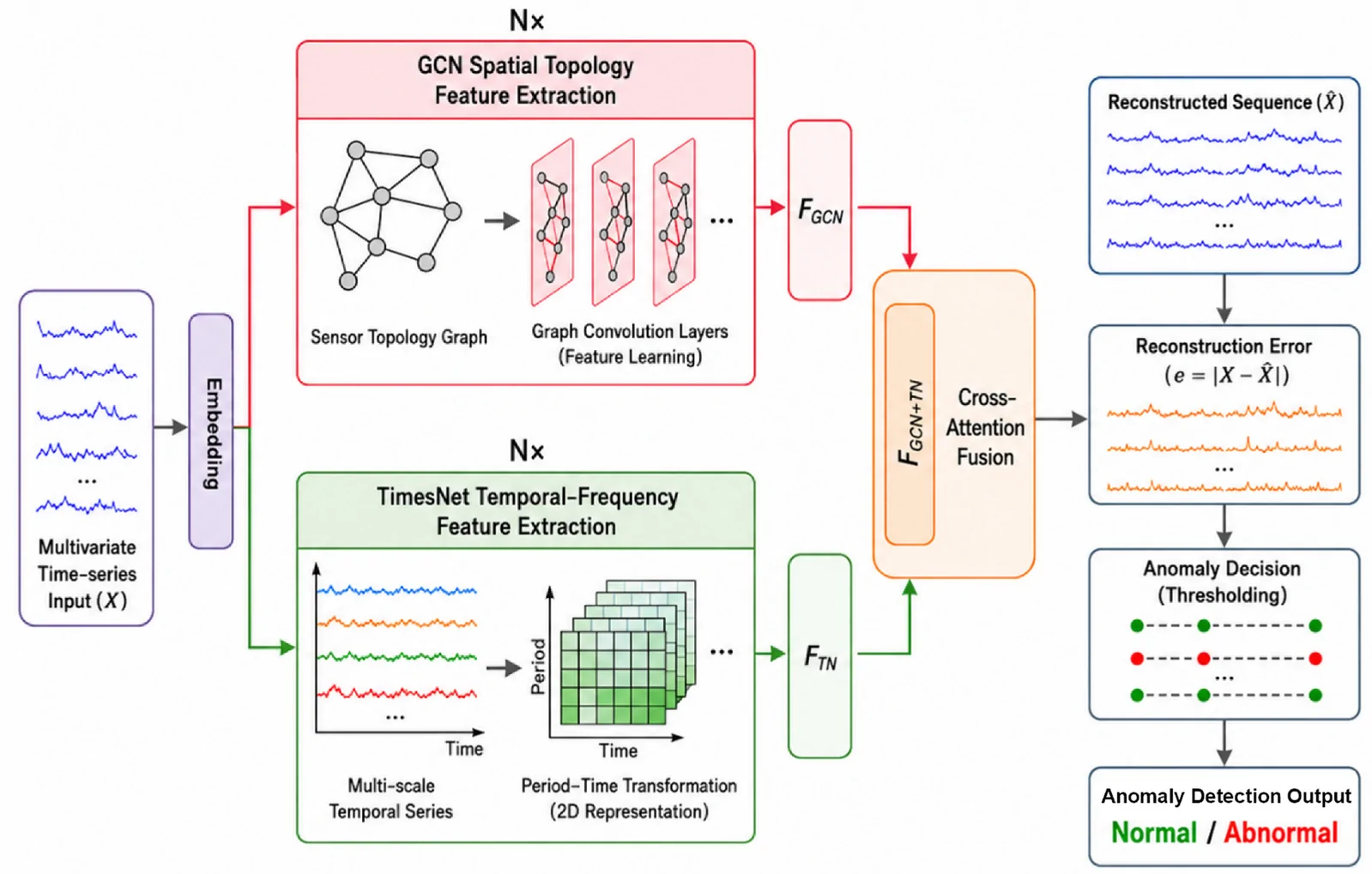
Overall framework of GasNet for precursor-related anomaly detection in underground mine sensor networks.

Based on the multi-source sensor-role hierarchy and the engineering-prior sensor graph, GasNet is designed to jointly model spatial–topological dependence, temporal–spectral variation, and adaptive feature fusion in underground mine sensor data. The input monitoring sequence is first organized as a graph-structured multivariate time series, where each node corresponds to a sensor variable and each edge represents a physically meaningful relationship defined by the engineering-prior graph. The GCN branch extracts spatial–topological correlations among sensor nodes, while the TimesNet branch captures non-stationary temporal–spectral patterns from the monitoring sequence. The two feature streams are then integrated through a cross-attention module. Finally, the fused representation is used for reconstruction-based anomaly detection, and monitoring intervals with large reconstruction deviations are regarded as precursor-related abnormal intervals. The overall framework of GasNet is shown in Figure 1.

#### 2.3.1. Spatial Dependence Modeling via GCN

A graph convolutional network (GCN) is used to aggregate information from neighboring sensor nodes according to the engineering-prior adjacency matrix. Different from a fully connected or unordered multivariate input, the graph structure constrains message passing along physically meaningful sensor relationships, such as ventilation-related propagation and equipment-induced coupling.

Before graph convolution, self-loops are added to the binary engineering-prior adjacency matrix, i.e., $\tilde{A}=A+I$. The resulting adjacency matrix is symmetrically normalized as

(4) $$\hat{A}={\tilde{D}}^{-\frac{1}{2}}\tilde{A}{\tilde{D}}^{-\frac{1}{2}},$$

where $\tilde{D}$ is the degree matrix of $\tilde{A}$. The layer-wise graph convolution is then expressed as

(5) $$H^{\left(l+1\right)}=\sigma \left(\hat{A}H^{\left(l\right)}W^{\left(l\right)}\right),$$

where $H^{\left(l\right)}$ denotes the node representation at layer l, $W^{\left(l\right)}$ is the trainable weight matrix, and σ(⋅) denotes the nonlinear activation function.

After the directed binary adjacency matrix, *A*, is constructed as described in Section 2.2, self-loops are first added to obtain $\tilde{A}=A+I$. The degree matrix, $\tilde{D}$, is then calculated from $\tilde{A}$, after which symmetric normalization is performed as $\hat{A}={\tilde{D}}^{-\frac{1}{2}}\tilde{A}{\tilde{D}}^{-\frac{1}{2}}$. The normalized matrix, $\hat{A}$, is used for GCN message passing.

#### 2.3.2. Temporal–Spectral Modeling via TimesNet

Underground mine monitoring signals usually exhibit non-stationary fluctuations and multi-scale periodic patterns caused by production cycles, ventilation adjustment, and equipment operation. To capture these temporal–spectral characteristics, the TimesNet backbone is introduced to transform one-dimensional time series into two-dimensional tensors according to dominant periods identified by the Fast Fourier Transform (FFT). For an input sequence, the amplitude of each frequency component is calculated as

(6) $$A_{f}=Amp(FFT(X)),$$

where FFT(⋅) denotes the Fourier transform and Amp(⋅) denotes amplitude extraction. The top-*k* dominant periods are selected according to the amplitude spectrum. The original one-dimensional sequence is then reshaped into two-dimensional tensors based on the selected periods and processed by TimesBlock to capture both intra-period and inter-period variations.

#### 2.3.3. Graph–Temporal–Spectral Fusion via Cross-Attention

The GCN branch and the TimesNet branch provide complementary information. The GCN branch focuses on spatial–topological dependencies among sensor nodes, whereas the TimesNet branch captures temporal–spectral variations in monitoring sequences. To adaptively integrate these two feature streams, a cross-attention mechanism is introduced instead of simple concatenation. Given the spatial–topological feature representation, $H_{g}$, and the temporal–spectral feature representation, $H_{t}$, the attention operation is defined as

(7) $$Attention(Q,K,V)=softmax\left(\frac{QK^{T}}{\sqrt{d_{k}}}\right)V,$$

where *Q*, *K*, and *V* denote the query, key, and value matrices, respectively, and $d_{k}$ is the dimension of the key vector. In GasNet, the two branches interact through a dual-way attention structure, allowing the fused representation to emphasize temporal–spectral variations that are supported by spatial–topological sensor relationships and vice versa. This adaptive fusion helps distinguish coordinated abnormal sensor responses from isolated short-term fluctuations. In GasNet, the spatial–topological representation generated by GCN and the temporal–spectral representation generated by TimesNet are used alternately as the Query and Key/Value inputs. This bidirectional cross-attention allows the fusion module to model both GCN-to-TimesNet and TimesNet-to-GCN dependencies. The resulting cross-attended representations are subsequently fused and mapped back to the original monitoring space for sequence reconstruction.

### 2.4. Reconstruction-Based Anomaly-Detection Strategy

Because real coal and gas outburst accident samples are extremely rare, GasNet adopts an unsupervised reconstruction-based anomaly-detection strategy. The model is trained using normal production data to learn the regular coupling patterns among methane-related, environmental, and operational sensor variables. During inference, a monitoring interval is regarded as abnormal when its reconstruction error significantly deviates from the normal-error distribution.

A key issue in multi-source underground mine monitoring is that not all sensor deviations have the same warning significance. For example, short-term fluctuations in shearer-operation variables or dust concentration may be caused by routine production activities and do not necessarily indicate gas-related abnormality. If the reconstruction errors of all sensors are treated equally, such auxiliary fluctuations may lead to unnecessary false alarms. Therefore, the training objective and anomaly scoring metric are decoupled in this study.

1. Training Objective: During training, GasNet reconstructs all monitoring variables so that the model can learn the complete coupling relationship between methane-related core sensors and environmental–operational modulation sensors. The global reconstruction loss is defined as
  (8) $$L_{\mathrm{rec}}=\frac{1}{TN}\sum_{t=1}^{T}\sum_{i=1}^{N}{\left(x_{t,i}-{\hat{x}}_{t,i}\right)}^{2},$$
  where $x_{t,i}$ and ${\hat{x}}_{t,i}$ denote the observed and reconstructed values of the *i*-th sensor at time step *t*, respectively; *T* is the input window length; and *N* is the number of sensor variables.
2. Sensor-role-aware anomaly scoring. During inference, the anomaly score is calculated by emphasizing reconstruction errors of methane-related core sensors while retaining a small contribution from modulation sensors. The sensor-role-aware anomaly score is defined as
  (9) $$S=\frac{1}{T}\sum_{t=1}^{T}\left(\frac{1}{\left|C\right|}\sum_{i\in C}|x_{t,i}-{\hat{x}}_{t,i}|+\lambda \frac{1}{\left|M\right|}\sum_{j\in M}|x_{t,j}-{\hat{x}}_{t,j}|\right),$$
  where *C* and *M* denote the sets of methane-related core sensors and modulation sensors, respectively, and λ is a weighting coefficient for modulation sensors. In this way, GasNet does not trigger warnings only because of isolated deviations in auxiliary operational or environmental variables. Instead, a high anomaly score is mainly produced when methane-related sensor responses cannot be well reconstructed under the learned normal environmental and operational context.
3. Threshold determination and interval adjustment. The anomaly threshold is determined from the reconstruction-error distribution of normal training data. A monitoring window is identified as abnormal when its anomaly score exceeds the threshold:
  (10) $$y=\left\{\begin{array}{ll} 1, & S>\theta \\ 0, & S\leq \theta \end{array}\right.,$$
  where y=1 denotes an abnormal window and y=0 denotes a normal window. To improve event-level continuity, an interval adjustment strategy is applied during evaluation. If at least one detected abnormal point overlaps with an annotated abnormal interval, the corresponding interval is regarded as detected. This strategy is used only for performance evaluation and does not change the model training process.

### 2.5. Multi-Level Interpretability Framework

To improve the engineering trustworthiness of GasNet, this study introduces a multi-level interpretability framework. The purpose of this framework is not to infer the actual physical source of an abnormal event, but to trace the model evidence that supports each warning. It contains two complementary levels: macroscopic feature-fusion interpretation and microscopic sensor-time contribution tracing.

The distillation procedure is performed strictly after completion of GasNet training. GasNet is first trained using the predominantly normal training set described in Section 3.2. After the teacher parameters are fixed, the same training-set samples are used to train the 1D-CNN student. Consistent with the unsupervised training assumption of GasNet, all samples in the training period are assigned a normal hard target (y = 0); no manually annotated abnormal labels are available or introduced during student training. Meanwhile, the fixed GasNet teacher generates continuous outputs for the same training samples, which are used as soft targets to transfer the teacher’s learned response behavior to the student. Thus, student training combines supervision toward the normal training state with teacher-derived soft supervision. The annotated abnormal intervals in the test period are not used to optimize either the teacher or the student model and are introduced only after training for evaluation.

The student model is optimized using a combination of a hard-target loss and a soft-target distillation loss:

(11) $$L_{KD}=(1-\alpha )L_{hard}+\alpha L_{soft},$$

where $L_{\mathrm{hard}}$ constrains the student prediction using the hard target constructed during the training-stage distillation procedure, $L_{\mathrm{soft}}$ encourages the student to approximate the output behavior of the fixed GasNet teacher, and α controls the contribution of the soft-target distillation term. The hard-target loss constrains the student toward the predominantly normal training-state assumption, whereas the soft-target loss encourages the student to approximate the finer-grained output behavior learned by GasNet.

After training, activation mapping is applied to the last convolutional layer of the student model. The resulting activation patterns are used to identify sensor channels and temporal positions that contribute strongly to the student warning output. The student model does not participate in GasNet training, anomaly-threshold determination, or the anomaly-detection results reported in Section 3; it serves only as a distilled interpretability model for subsequent sensor-time tracing.

The two interpretability levels provide complementary model evidence. Cross-attention analysis characterizes how GasNet balances spatial–topological and temporal–spectral representations at the feature-fusion level, whereas the distilled 1D-CNN provides heuristic sensor-time surrogate evidence for subsequent warning review. Neither attention weights nor student activation maps are interpreted as direct physical causality; instead, they are used together with ablation results and field monitoring information for engineering verification.

## 3. Case Study and Experimental Results

### 3.1. Study Area and Sensor Deployment

The field case was conducted at the Xinyuan Coal Mine, located in Jinzhong City, Shanxi Province, China. The 31002 fully mechanized working face was selected as the study area because it is located in a high-gas mine with complex gas-geological conditions. According to three-dimensional seismic exploration, in-seam radio-wave penetration, and actual geological records, the working face was expected to contain several concealed geological structures, including 24 faults, five collapse columns, one erosion zone, and five anomalous bodies. These geological conditions may affect gas occurrence, gas emission, and sensor responses during mining, making this working face suitable for validating precursor-related anomaly detection using underground mine sensor data.

**Figure 2:**
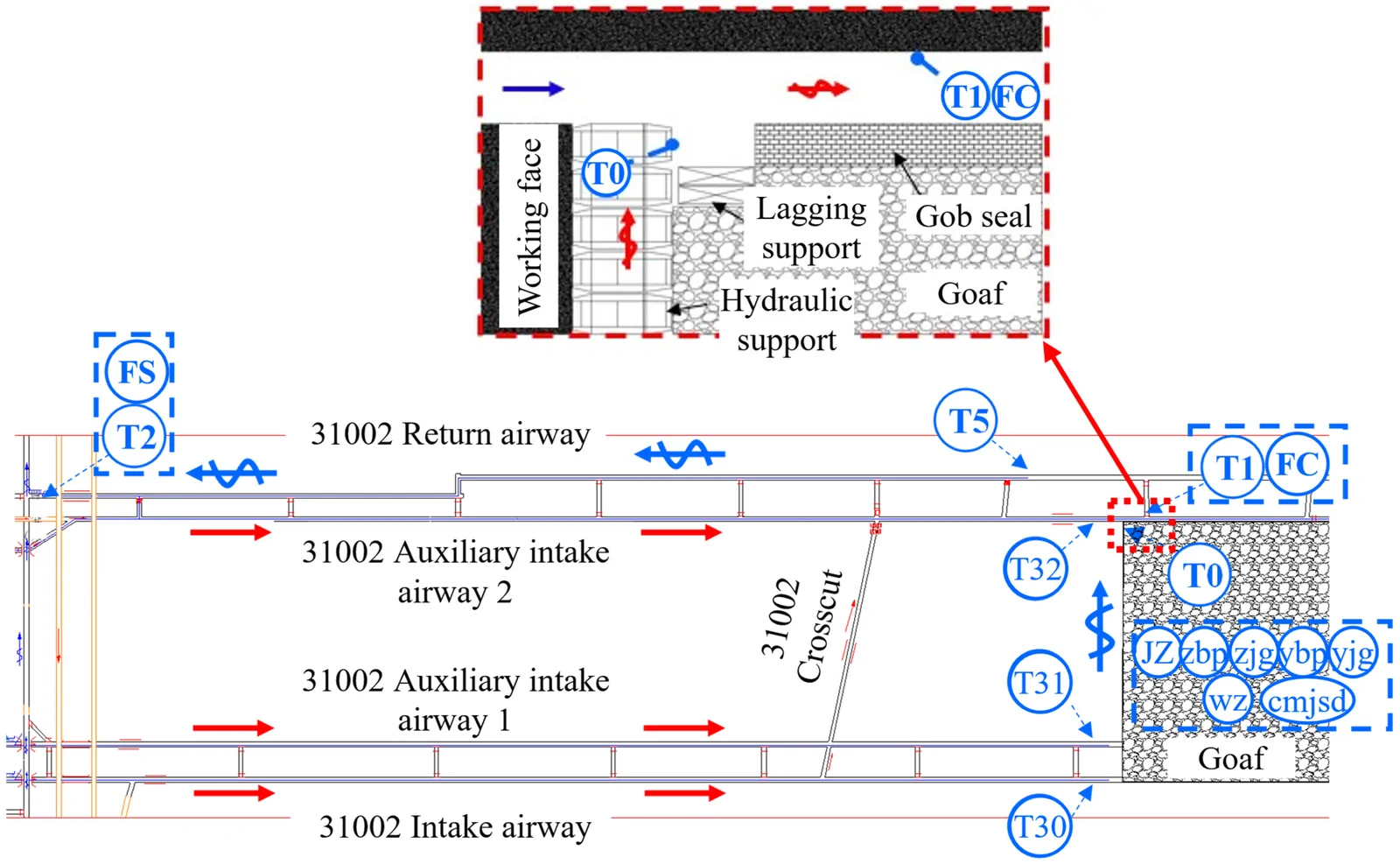
Sensor deployment and ventilation layout of the 31002 working face.

The 31002 working face adopts a “three-intake and one-return” ventilation layout and a W-type ventilation mode. During retreat mining, four gate roads were arranged, namely, the 31002 intake roadway, the 31002 auxiliary intake roadway 1, the 31002 auxiliary intake roadway 2, and the 31002 return airway. The 31002 auxiliary intake roadway 2 was constructed using a gob-side entry retaining technique. The working face was arranged along the strike direction, and the intake roadway, auxiliary intake roadways, return airway, and open-off cut were all arranged along the coal seam roof. The layout of the working face, ventilation route, and monitoring sensor deployment is shown in Figure 2.

The monitoring data used in this study were obtained from the KJ90X mine safety monitoring system produced by the China Coal Technology and Engineering Group Chongqing Research Institute Co., Ltd., (Chongqing, China) and the MG500/1130-WD3 AC electric traction shearer system (Xi’an Coal Mining Machinery Co., Ltd., Xi’an, China). The selected sensor variables include methane concentration, wind velocity, dust concentration, and shearer-operation variables. Specifically, the methane-related sensors include T30, T31, T32, T0, T1, T5, and T2 and the onboard methane sensor of the shearer, denoted as JZ. The environmental sensors include the wind velocity sensor, FS, and the dust concentration sensor, FC. The shearer-operation variables include traction speed (cmjsd), left frequency-conversion output current (zbp), left cutting motor current (zjg), right frequency-conversion output current (ybp), right cutting motor current (yjg), and shearer position (wz). These variables jointly describe methane emission and accumulation, ventilation dilution, coal fragmentation, and mining disturbance.

The monitoring data from 00:00:00 on 1 January 2022 to 23:59:45 on 13 May 2022 were selected as the sample source. To ensure temporal consistency among heterogeneous sensor streams, the raw records were resampled to a unified interval of 15 s. Within each 15 s window, the average value of each sensor variable was calculated and stored, thereby reducing the influence of instantaneous disturbances and occasional missing records. The detailed preprocessing procedure, train–test split, and abnormal interval annotation strategy are described in Section 3.2.

### 3.2. Data Preprocessing and Abnormal Interval Annotation

To ensure temporal consistency among heterogeneous sensor streams, the raw multi-source records were first resampled to a unified interval of 15 s. The 15 s sequence was used as the common temporally aligned data source for subsequent experiments. For the main comparative and ablation experiments, the aligned sequence was further aggregated to a 5 min sampling interval. The 1 min and 3 min versions were generated only for the sampling-interval sensitivity analysis in Section 3.6. Therefore, unless otherwise stated, the experimental results reported in this study correspond to the 5 min sampling configuration. For each 15 s window, the average value of each sensor variable was calculated and stored. This operation reduces the influence of instantaneous fluctuations and occasional missing records while preserving the dynamic evolution characteristics of the monitoring sequence. After resampling, all sensor variables were aligned according to timestamps to form a multivariate time-series dataset.

For data quality control, calibration-induced methane anomalies were removed before model training. In the original monitoring data, periodic calibration operations occurred approximately every 15 days, during which methane readings could rise above 1% within a short period. These artificial peaks do not reflect actual gas emission from the working face and may introduce false abnormal patterns. Therefore, calibration-induced abnormal segments were identified according to monitoring records, removed from the original sequence, and recovered by linear interpolation. No large-scale missing records were observed during the study period.

The dataset was then divided chronologically to avoid temporal leakage, with an approximate training–test ratio of 8:2. The data from 1 January 2022 to 14 April 2022 were used for model training, while the data from 15 April 2022 to 13 May 2022 were used for testing. Because GasNet follows an unsupervised reconstruction-based anomaly-detection paradigm, abnormal-event labels were not used during model training. The training period (1 January to 14 April 2022) was treated as predominantly normal production data and was used to learn the dominant spatio-temporal coupling patterns among methane-related, environmental, and operational variables. Unlike the test period, the training period was not systematically annotated for precursor-related abnormal events. Therefore, a small number of unrecorded or weak abnormal segments may remain in the training data. Nevertheless, no actual coal and gas outburst occurred during the study period, and obvious artificial anomalies caused by sensor calibration were removed during preprocessing. The model was consequently trained to characterize the dominant production-state distribution rather than an artificially purified set of exclusively normal samples. The possible presence of a small proportion of unidentified abnormal segments in the training set is acknowledged as a limitation of the present field study. During testing, precursor-related abnormal intervals were annotated according to field records to evaluate the model’s ability to identify previously unseen abnormal monitoring segments. These abnormal-interval labels were used exclusively as ground truth for test-stage performance evaluation and the point-adjustment procedure described in Section 3.3; they were not used for GasNet training, reconstruction-loss optimization, or anomaly-threshold determination. All variables were standardized using the mean and standard deviation calculated from the training set only, and the same parameters were applied to the test set. Given the standardized multivariate sequence, sliding windows were constructed with a window length of 100 time steps.

It should be emphasized that the abnormal labels in this study are not actual coal and gas outburst accident labels. No real outburst accident occurred during the selected monitoring period. Instead, abnormal intervals were defined as precursor-related abnormal intervals according to field monitoring anomalies and operational records. The annotation criteria mainly included methane over-limit events; abnormal drilling phenomena such as drill sticking, drill jamming and gas spraying from boreholes; difficult excavation; exposure of geological anomalies such as faults and collapse columns; and the addition of gas drainage or discharge boreholes. These phenomena are usually associated with coal fragmentation, abnormal gas occurrence, geological-structure disturbance, or enhanced local gas release, and they are important precursor information used in outburst-prevention engineering to judge potential abnormal states.

Based on these criteria, the test period was annotated into normal intervals and precursor-related abnormal intervals. The occurrence times of the abnormal events were obtained from contemporaneous field records provided by technical personnel of the cooperating mine rather than being determined retrospectively from the sensor curves or model outputs. After the field-recorded events had been identified, a predefined temporal rule was applied to construct the evaluation labels: the 1 h period preceding each recorded event was designated as a precursor-related abnormal interval. Accordingly, eight abnormal intervals were identified during the test period: 15 April, 00:40–01:40; 7 May, 11:30–12:30 and 13:05–14:05; 8 May, 11:05–12:05; and 10 May, 12:10–13:10, 14:00–15:00, 21:05–22:05, and 10 May, 23:35–11 May, 00:35.

The field records used to identify these events documented precursor-related or engineering abnormal conditions, including methane over-limit events; abnormal drilling phenomena such as drill sticking, drill jamming and gas spraying from boreholes; difficult excavation; geological anomaly exposure such as faults and collapse columns; and additional gas drainage or discharge measures. The retained records, however, do not permit a reliable one-to-one assignment of these specific event types to each of the eight individual timestamps; therefore, no retrospective event-specific categories were introduced in this study.

The original abnormal-event records were provided by mine technical personnel, and the authors generated the corresponding 1 h evaluation intervals using the predefined temporal rule. The intervals were not independently re-annotated by multiple experts, and therefore no inter-rater agreement statistic was available. Because the interval boundaries were generated using a fixed rule rather than manually adjusted according to the sensor trajectories or model predictions, this procedure reduces, but does not eliminate, potential annotation subjectivity. The absence of independent multi-expert annotation is acknowledged as a limitation of the present study.

### 3.3. Evaluation Metrics and Experimental Settings

**Figure 3:**
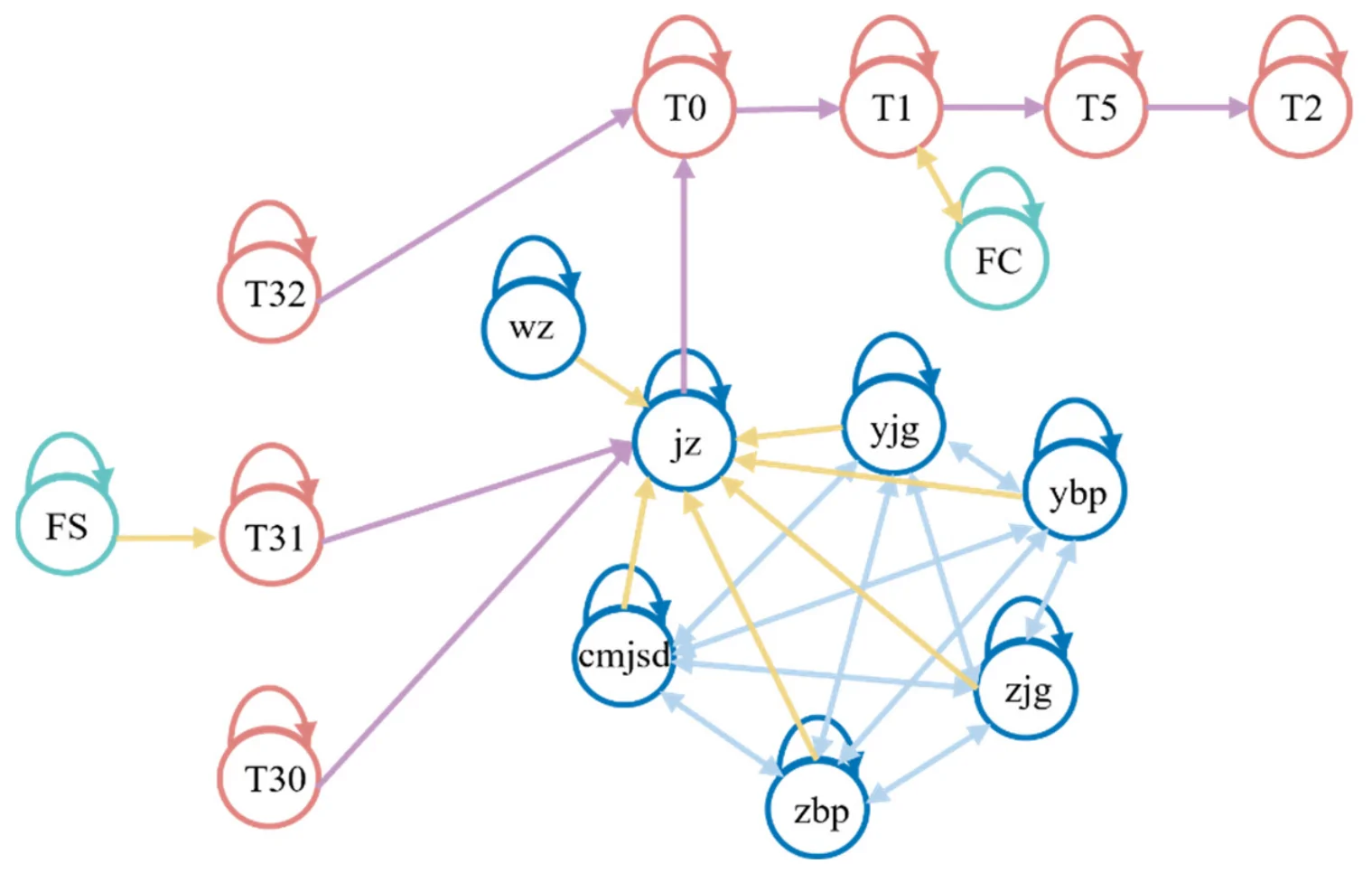
Schematic diagram of sensor-node relationships in the engineering-prior graph. Note: Directed arrows indicate the specified engineering relationships between sensor nodes; node labels correspond to the sensor codes listed in Table 2.

For the 31002 working face, the engineering-prior sensor graph used by the GCN branch was constructed according to the graph construction rules described in Section 2.2 and the actual sensor deployment described in Section 3.1. The graph nodes correspond to the selected sensor variables, including methane-related sensors, wind velocity, dust concentration, and shearer-operation variables. Directed or coupled edges were established according to ventilation causality, equipment disturbance, and physically meaningful sensor relationships. Following these principles, the fixed methane-monitoring pathway was represented mainly by T32 → T0 → T1 → T5 → T2. T30 and T31 were connected to the onboard methane sensor JZ according to their relative upstream–downstream positions, while JZ → T0 linked the moving shearer-related sensing subsystem to the fixed methane-monitoring pathway. The shearer-operation variables cmjsd, zbp, zjg, ybp, and yjg were connected bidirectionally within the equipment-operation subsystem and were further connected to JZ, while the shearer-position variable, wz, was connected to JZ. For the environmental variables, FS → T31 represented the local ventilation-related connection, whereas FC and T1 were connected bidirectionally according to their spatial proximity and coupled responses near the working face. The resulting sensor-node relationship is shown in Figure 3, and the corresponding adjacency matrix used in the GCN structure is presented in Table 2.

It should be noted that the adjacency matrix was not obtained by purely data-driven correlation screening. Instead, the ventilation route, roadway layout, methane sensor locations, and shearer-related coupling relationships were first used to determine the main graph structure, while statistical correlations were only used as supplementary evidence. This design ensures that graph convolution follows physically meaningful information pathways during feature aggregation.

To evaluate precursor-related anomaly-detection performance, six metrics were used: Precision, Recall, F1-score, false-alarm rate (FAR), false-negative rate (FNR), and the number of false positives (FP). To account for the segment-wise nature of precursor-related abnormal events, a point-adjustment strategy was applied during evaluation. This procedure was used only for performance assessment and did not affect model training, anomaly-score generation, or threshold determination. Specifically, if at least one predicted abnormal point overlapped with an annotated abnormal interval, the predicted normal points within that same ground-truth interval were adjusted to abnormal so that a successfully detected continuous event was not penalized as multiple missed points. After this adjustment, all TP, FP, FN, and TN values were counted at the point level. Accordingly, Precision, Recall, F1-score, FAR, FNR, and FP as reported in this study are all point-level metrics calculated from the same point-adjusted prediction sequence. Therefore, the evaluation does not combine event-level true positives with point-level false positives. The annotated intervals are used only during the evaluation-stage point-adjustment procedure to account for the continuity of abnormal events. Because this procedure uses the annotated interval boundaries to adjust predictions within successfully detected abnormal intervals, the resulting Recall and F1-score should be interpreted as point-level metrics after continuity adjustment rather than as raw online point-wise detection performance. To avoid confusion, the previous statement that an abnormal interval was simply “regarded as detected” has been revised accordingly. The metric definitions are as follows:

(12) $$Precision=\frac{TP}{TP+FP},$$

(13) $$Recall=\frac{TP}{TP+FN},$$

(14) $$F1=2\times \frac{Precision\times Recall}{Precision+Recall},$$

(15) $$FAR=\frac{FP}{TN+FP},$$

(16) $$FNR=\frac{FN}{TP+FN},$$

Here, *TP* denotes abnormal samples correctly identified as abnormal, *FP* denotes normal samples incorrectly identified as abnormal, *FN* denotes abnormal samples missed by the model, and *TN* denotes normal samples correctly identified as normal. *Precision* reflects the reliability of warning outputs, *Recall* reflects the ability to detect annotated abnormal intervals, and *F1-score* measures the balance between Precision and Recall. *FAR* describes the false-alarm level under normal production conditions, while *FNR* indicates the missed-warning risk. In underground mine safety monitoring, Recall and FNR are particularly important because missed abnormal intervals may lead to delayed engineering response, whereas FAR and FP reflect the disturbance caused by unnecessary alarms.

GasNet was compared with seven representative time-series models: Transformer, Autoformer, Crossformer, DLinear, FEDformer, FiLM, and Informer. These baselines were selected to cover complementary temporal-modeling paradigms rather than multiple variants of a single architecture. Transformer provides a standard self-attention reference; Informer represents efficient long-sequence attention modeling; Autoformer introduces decomposition and autocorrelation mechanisms; FEDformer performs frequency-enhanced decomposition; Crossformer models cross-variable and cross-scale temporal dependencies; DLinear provides a lightweight decomposition-based linear baseline; and FiLM represents frequency-domain long-term time-series modeling. Together, these models provide a diverse benchmark for examining whether the proposed engineering-prior graph–temporal–spectral fusion offers additional value beyond temporal modeling alone. In addition, graph-related comparisons are provided through the GCN-only and prior-graph ablation models in Section 3.5, which directly examine the contribution of graph representation and engineering-prior topology. The present benchmark is therefore intended to compare GasNet with representative temporal modeling architectures and its own graph-based ablations, rather than to constitute an exhaustive survey of all existing time-series anomaly-detection methods.

All forecasting-oriented baselines were adapted to the same prediction/reconstruction-error-based anomaly-detection pipeline. The same training–test split, sampling interval, sliding-window configuration, and evaluation procedure were used across models. Hyperparameters were selected within architecture-appropriate ranges according to training convergence and stable prediction/reconstruction loss rather than by maximizing performance on the annotated test events.

For a consistent anomaly-detection comparison, all forecasting-oriented baseline models were adapted to the same reconstruction/prediction-error-based detection framework. Each model received the same multivariate sliding-window input and generated an estimate of the target monitoring state. The absolute reconstruction or prediction error was then converted into an anomaly score using the same evaluation pipeline as GasNet, and the anomaly threshold was determined from the corresponding training-data error distribution. Thus, the comparison focuses on differences in feature-representation capability rather than differences in anomaly-decision rules.

To ensure comparable tuning effort, the principal hyperparameters of all models were selected within commonly used ranges while keeping the input window, sampling interval, training–test split, optimizer family, and anomaly-thresholding procedure consistent. Model dimensions were controlled to remain at a comparable scale whenever the architecture permitted. The final settings were selected based on training convergence and stable reconstruction/prediction loss rather than by maximizing performance on the annotated test events, thereby avoiding direct test-set tuning.

Unless otherwise stated, the main comparative and ablation experiments used a sampling interval of 5 min and an input window length of 100 time steps. Therefore, each input sample contains approximately 500 min (8 h 20 min) of historical multi-source monitoring information. This window length represents the temporal context available to GasNet for each anomaly decision rather than the warning latency of the system. For GasNet, the GCN branch consisted of two graph-convolution layers with an output dimension of 32 and ReLU activation. The TimesNet branch contained three TimesBlocks, with $d_{\mathrm{mod}el}=64$, $d_{ff}=64$, and $c_{out}=128$. The outputs of the GCN and TimesNet branches were projected to a common latent dimension of 64 before single-head bidirectional cross-attention fusion. A dropout rate of 0.1 was used in the GCN, TimesNet, and feature-fusion modules. The fused representation was mapped to the original monitoring space through a linear reconstruction layer. The modulation-variable weighting coefficient in the anomaly score was set to λ=0.1.

GasNet was optimized using Adam with an initial learning rate of 0.001 and a batch size of 32. The maximum number of training epochs was set to 50. Early stopping with a patience of 10 epochs was applied according to the reconstruction loss, and no additional learning-rate scheduler was used. The random seed was fixed at 42 for NumPy and PyTorch. The experiments were implemented using PyTorch (v2.6.0) and DGL (v2.4.0) on a workstation running Windows 11 with an Intel Core i5-12600KF CPU (3.70 GHz) and an NVIDIA RTX 3090 GPU with 24 GB memory.

For reconstruction-based anomaly detection, the decision threshold was determined exclusively from the anomaly-score distribution of the training data and was not optimized using the annotated test events. Specifically, the threshold was defined as τ=μ+3σ, where μ and σ denote the mean and standard deviation of the anomaly scores obtained from the training data, respectively. This distribution-based strategy avoids using test-set labels for threshold calibration. The robustness of this choice was further examined by comparing thresholds of μ+σ, μ+2σ, and μ+3σ, as reported in Section 3.6.

### 3.4. Comparison with Baseline Models

The comparative results of GasNet and the seven baseline models are summarized in Table 3. Overall, all baseline models show a certain capability in detecting precursor-related abnormal intervals from multi-source sensor data, but their performance differs substantially in terms of missed detections and false alarms.

Under the point-adjusted evaluation protocol used in this study, GasNet achieved the highest numerical F1-score among the compared models on the present dataset. This result indicates that GasNet successfully detects all annotated abnormal intervals in the test period while maintaining a low false-alarm level. Compared with Informer, the strongest baseline in the present comparison, GasNet shows a numerical increase in F1-score from 0.921 to 0.937 and reduces the number of false positives from 18 to 14. Although DLinear obtains a slightly lower FAR of 0.0014 and fewer false positives, its Recall is only 0.750, indicating that it misses part of the annotated abnormal intervals. This result illustrates the trade-off between false-alarm suppression and abnormal-interval coverage: although DLinear produces fewer false positives, its lower Recall indicates a higher missed-detection risk in the present test set.

Transformer and Informer both achieve a Recall of 1.000, showing good sensitivity to abnormal sensor intervals. However, Transformer produces 35 false positives and an FAR of 0.0042, indicating that it is overly sensitive to normal production fluctuations. Informer performs more stably, with an FAR of 0.0021 and 18 false positives, but its Precision and F1-score remain lower than those of GasNet. These results indicate that long-sequence attention models can capture abnormal temporal patterns effectively. Their higher false-positive counts relative to GasNet may partly reflect the absence of explicit sensor-topology and engineering-context constraints; however, this interpretation should be considered together with the ablation results in Section 3.5 rather than inferred from the baseline comparison alone.

Autoformer, Crossformer, and FEDformer show intermediate performance. Their Recall values are all 0.875, corresponding to an FNR of 0.125, which means that each model misses one annotated abnormal interval. Their Precision values range from 0.805 to 0.819, and their F1-scores range from 0.838 to 0.846. These results indicate that decomposition-based and frequency-enhanced time-series models can capture part of the abnormal temporal evolution, but they are less effective in identifying weak or boundary abnormal intervals under complex underground monitoring conditions. FiLM shows the weakest comprehensive performance, with an F1-score of 0.774, a Recall of 0.750, and 33 false positives.

The superior performance of GasNet can be attributed to its engineering-prior graph–temporal–spectral fusion design. The engineering-prior sensor graph enables the GCN branch to aggregate information along ventilation-related and equipment-coupling pathways, reducing the influence of isolated sensor fluctuations. The TimesNet branch captures non-stationary temporal–spectral patterns in multi-source monitoring sequences, while cross-attention adaptively integrates spatial–topological and temporal–spectral information. As a result, GasNet achieves a better balance between detection sensitivity and false-alarm suppression, which is essential for precursor-related anomaly detection in underground mine sensor networks.

The performance differences between GasNet and the strongest baselines should be interpreted cautiously. In particular, GasNet and Informer both achieved complete coverage of the eight annotated abnormal intervals, while their principal difference was in false-alarm control. GasNet reduced the number of false positives from 18 to 14 and increased the F1-score from 0.921 to 0.937. Given the limited number of annotated abnormal events and the absence of repeated independent training runs in the original experiment, these differences are reported as numerical performance differences rather than as statistically significant improvements. A more rigorous statistical comparison based on repeated runs and larger event sets will be required to establish the significance and stability of these gains.

### 3.5. Ablation Study

To further verify the contribution of each component in GasNet, ablation experiments were conducted from three perspectives: spatial–topological modeling, temporal–spectral modeling, and engineering-prior sensor graph construction. Four ablated models were compared with the complete GasNet model: GCN-only, TimesNet-only, GasNet without the prior adjacency matrix, and GCN-only without the prior adjacency matrix. The comparative results are shown in Table 4.

The complete GasNet model achieves the best overall performance, with a Precision of 0.8814, a Recall of 1.0000, an F1-score of 0.9361, an FAR of 0.0017, an FNR of 0, and 14 false positives. This result confirms that the combination of spatial–topological modeling, temporal–spectral modeling, and cross-attention fusion is necessary for reliable precursor-related anomaly detection. By contrast, the GCN-only model maintains a relatively high Recall of 0.8750 but has a low Precision of 0.4375 and a large number of false positives. This indicates that spatial–topological information alone is sensitive to abnormal intervals but insufficient to suppress false alarms caused by normal production fluctuations.

The TimesNet-only model shows the opposite tendency. Its Precision reaches 0.6190, but the Recall decreases to 0.3750, and the F1-score is only 0.4684. This suggests that temporal–spectral features alone can capture part of the non-stationary fluctuation patterns but may miss spatially coordinated abnormal responses across multiple sensors. Therefore, relying only on temporal–spectral modeling is inadequate for underground mine sensor anomaly detection, especially when abnormal responses are weak or distributed across several monitoring locations.

The role of the engineering-prior adjacency matrix is particularly evident. When the prior adjacency matrix is removed from GasNet, the F1-score decreases from 0.9361 to 0.6667, the Recall decreases from 1.0000 to 0.6250, and the FNR increases from 0 to 0.3750. Meanwhile, false positives increase from 14 to 26. These changes show that the engineering-prior sensor graph is not merely an auxiliary structure; it provides essential spatial constraints for message passing and helps the model distinguish physically meaningful multi-sensor responses from unrelated fluctuations.

For the GCN-only branch, removing the prior adjacency matrix reduces false positives from 117 to 48 and improves Precision from 0.4375 to 0.5740, but Recall drops from 0.8750 to 0.6250 and FNR increases from 0.1250 to 0.3750. This result indicates that weakening the graph constraint may reduce some false alarms, but it also substantially increases missed abnormal intervals. For underground safety monitoring, missed abnormal intervals are generally more unacceptable than a moderate number of false alarms. Therefore, the prior adjacency matrix is more valuable when it is combined with temporal–spectral modeling and cross-attention fusion in the complete GasNet framework.

The substantially larger number of false positives produced by the GCN-only model (117) compared with GasNet without the prior adjacency matrix (26) further illustrates the complementary roles of graph constraints and temporal–spectral fusion. Although GCN-only retains the engineering-prior graph, it lacks the TimesNet branch and cross-attention fusion and therefore relies mainly on spatial–topological aggregation. Consequently, transient production-induced fluctuations that are spatially correlated across several sensors may be propagated and amplified by the graph structure, leading to excessive false alarms. In contrast, GasNet without the prior adjacency matrix still retains temporal–spectral modeling and cross-attention fusion, which provide additional temporal context and help suppress short-lived fluctuations that are not consistently supported across feature domains. This result suggests that the engineering-prior graph primarily contributes to abnormal-event coverage and missed-detection reduction, whereas temporal–spectral modeling and cross-attention also play an important role in false-alarm suppression.

The comparison between GasNet and GasNet without the prior adjacency matrix represents a large-scale topology perturbation that evaluates the functional relevance of the engineering-prior structure, rather than a fine-grained robustness test against small edge uncertainties. In the latter model, the sparse engineering-prior topology is replaced by a fully connected graph while the main graph–temporal–spectral fusion architecture is retained. This represents a substantial increase in graph density and removes the selective engineering constraints imposed by ventilation pathways, sensor deployment, and equipment-coupling relationships.

Under this topology perturbation, the F1-score decreases from 0.9361 to 0.6667, Recall decreases from 1.0000 to 0.6250, and false positives increase from 14 to 26. A similar trend can be observed in the GCN-only comparison, where replacing the engineering-prior graph with a fully connected graph reduces Recall from 0.8750 to 0.6250. These results indicate that GasNet does not benefit simply from increasing the number of graph connections. Instead, its performance depends on selectively preserving physically meaningful sensor relationships and suppressing unrestricted message propagation among unrelated nodes.

Therefore, the present ablation provides evidence that the engineering-prior topology is functionally important, but it does not establish robustness to small errors or uncertainties in individual graph edges. Fine-grained edge deletion, addition, and mechanism-specific perturbation experiments remain necessary for such robustness assessment.

**Figure 4:**
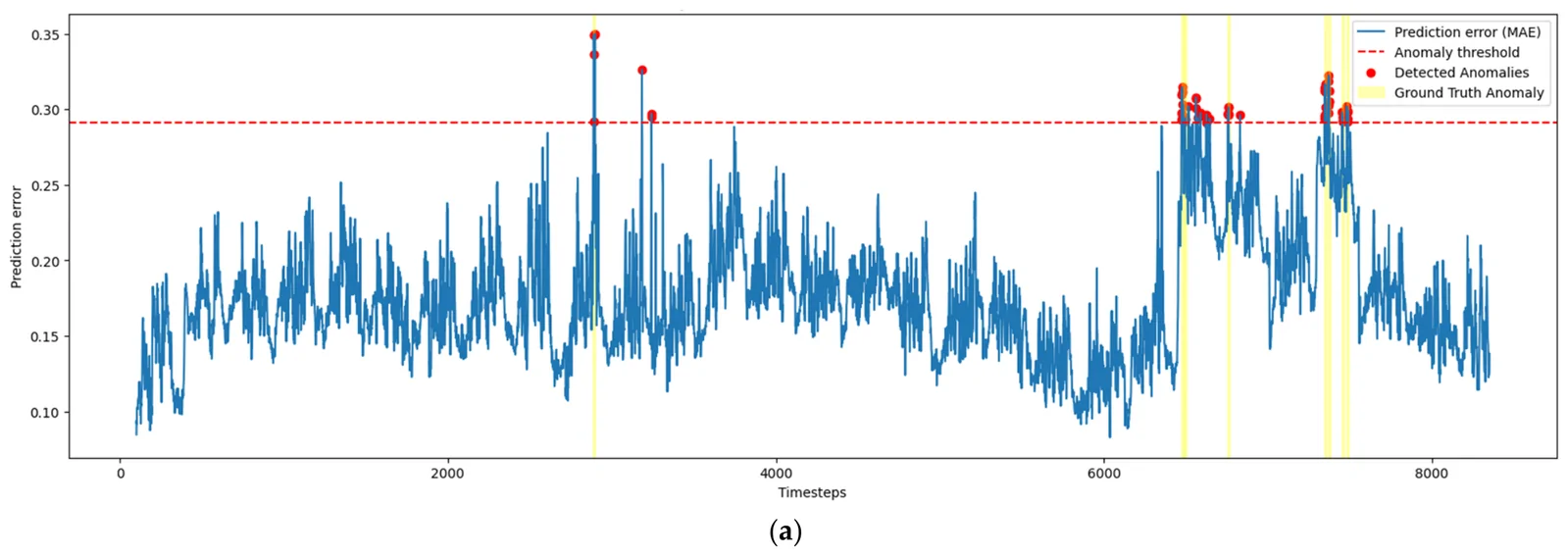

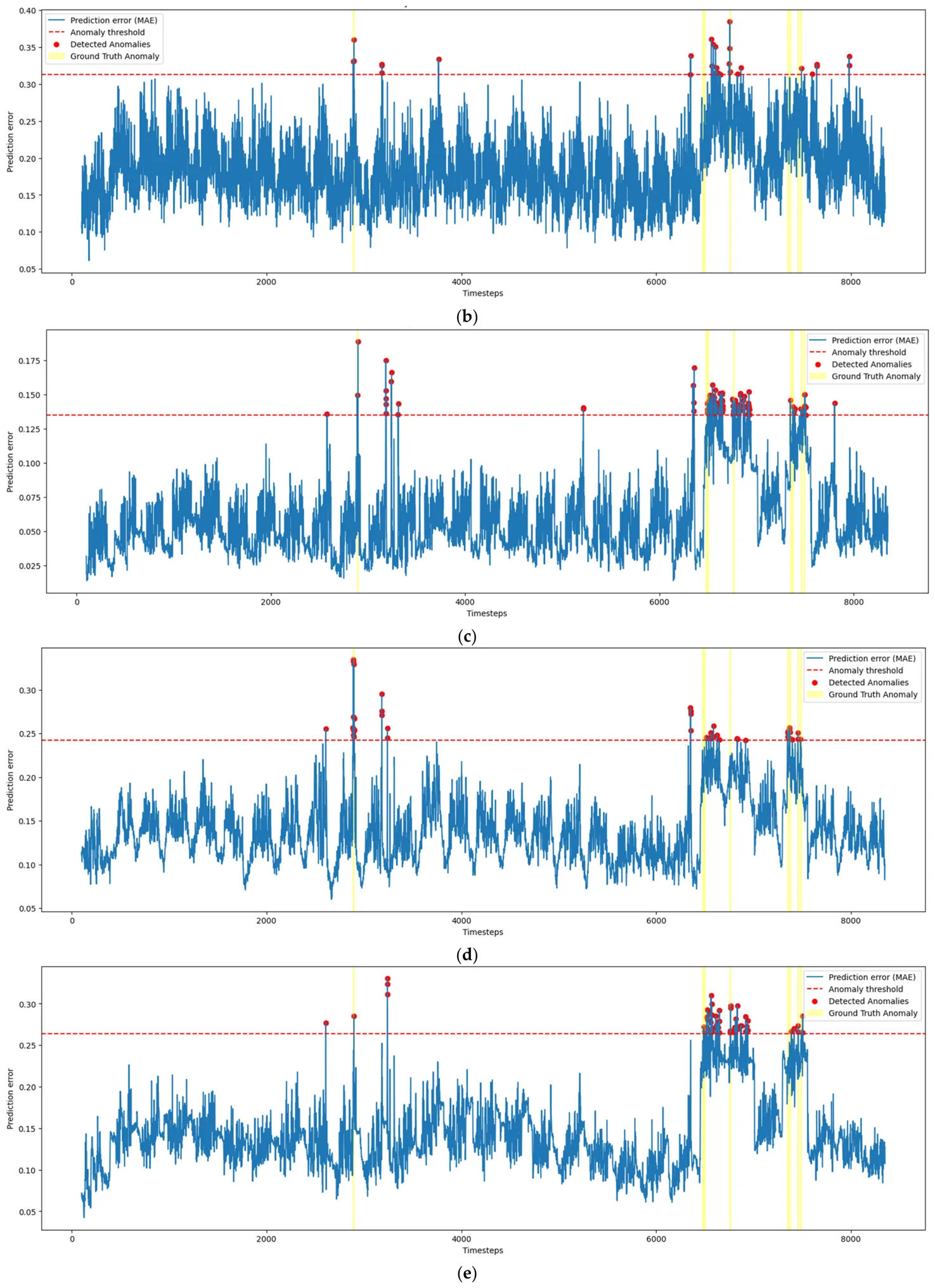
Visualization results of precursor-related anomaly detection by different ablated models: (**a**) GasNet; (**b**) GCN-only; (**c**) TimesNet-only; (**d**) GasNet without prior adjacency matrix; and (**e**) GCN without prior adjacency matrix.

To further examine the response behavior of different model structures, the anomaly-detection results of the ablated models are visualized in Figure 4. In the figure, the reconstruction-error curve reflects the deviation between the input sequence and the reconstructed sequence, the threshold line indicates the anomaly-decision boundary, the detected points represent model-triggered abnormal samples, and the shaded regions denote the annotated precursor-related abnormal intervals.

As shown in Figure 4a, GasNet produces clear error responses around most annotated abnormal intervals, and the detected abnormal points are highly consistent with the labeled intervals. Meanwhile, the reconstruction error remains relatively stable during most normal production periods, indicating that GasNet can suppress ordinary short-term disturbances while maintaining high sensitivity to precursor-related abnormal patterns. The GCN-only model in Figure 4b also responds to several abnormal intervals, but the detected points are widely scattered during normal periods, showing that spatial–topological modeling alone is sensitive but prone to false alarms. By contrast, the TimesNet-only model in Figure 4c produces fewer effective triggers around some abnormal intervals, suggesting that temporal–spectral modeling without spatial constraints may miss coordinated multi-sensor abnormal responses.

Figure 4d,e show the results after removing the prior adjacency matrix from GasNet and GCN-only, respectively. Although these models can still detect part of the abnormal intervals, their error peaks are less concentrated and their detected points show more boundary misjudgments or missed triggers. This visual comparison further confirms that the engineering-prior graph improves the focusing ability of anomaly detection by constraining information aggregation along physically meaningful sensor relationships.

Overall, the ablation results demonstrate that GCN contributes to spatially correlated anomaly perception, TimesNet contributes to non-stationary temporal–spectral representation, and cross-attention enables effective fusion of the two complementary feature streams. The engineering-prior adjacency matrix further enhances the reliability of spatial feature aggregation by embedding ventilation causality, sensor layout, and equipment-coupling relationships. These components jointly explain why GasNet achieves high Recall while maintaining a low false-alarm level. It should also be noted that reconstruction errors exceeding the threshold do not indicate that an outburst will necessarily occur; instead, they indicate that the multi-source sensor relationship in the current monitoring segment has significantly deviated from the learned normal production pattern.

### 3.6. Hyperparameter Sensitivity Analysis

To examine the robustness of GasNet under different experimental settings, sensitivity analyses were conducted for three key factors: detection window length, sampling interval, and anomaly threshold. These parameters respectively affect the temporal context used by the model, the resolution of monitoring signals, and the strictness of abnormal decision-making.

In addition to these data- and decision-level parameters, the sensitivity of model performance to a major topology change was examined through the graph ablation experiment in Section 3.5, where the engineering-prior graph was replaced by a fully connected graph. This experiment evaluates the importance of the prior topology but does not constitute a fine-grained robustness analysis of individual edge uncertainty.

The remaining architectural hyperparameters, including GCN depth, the latent attention dimension, the hidden dimension, and the modulation-variable reconstruction weight (λ), were fixed according to the implementation configuration reported in Section 3.3. Because the available field dataset contains only eight annotated abnormal intervals, an exhaustive multidimensional hyperparameter search would increase the risk of overfitting the limited test evidence. Therefore, the present study focuses the quantitative sensitivity analysis on window length, sampling interval, anomaly threshold, and the engineering-prior versus fully connected graph comparison. More systematic architectural sensitivity analyses will be conducted using larger multi-mine datasets.

**Figure 5:**
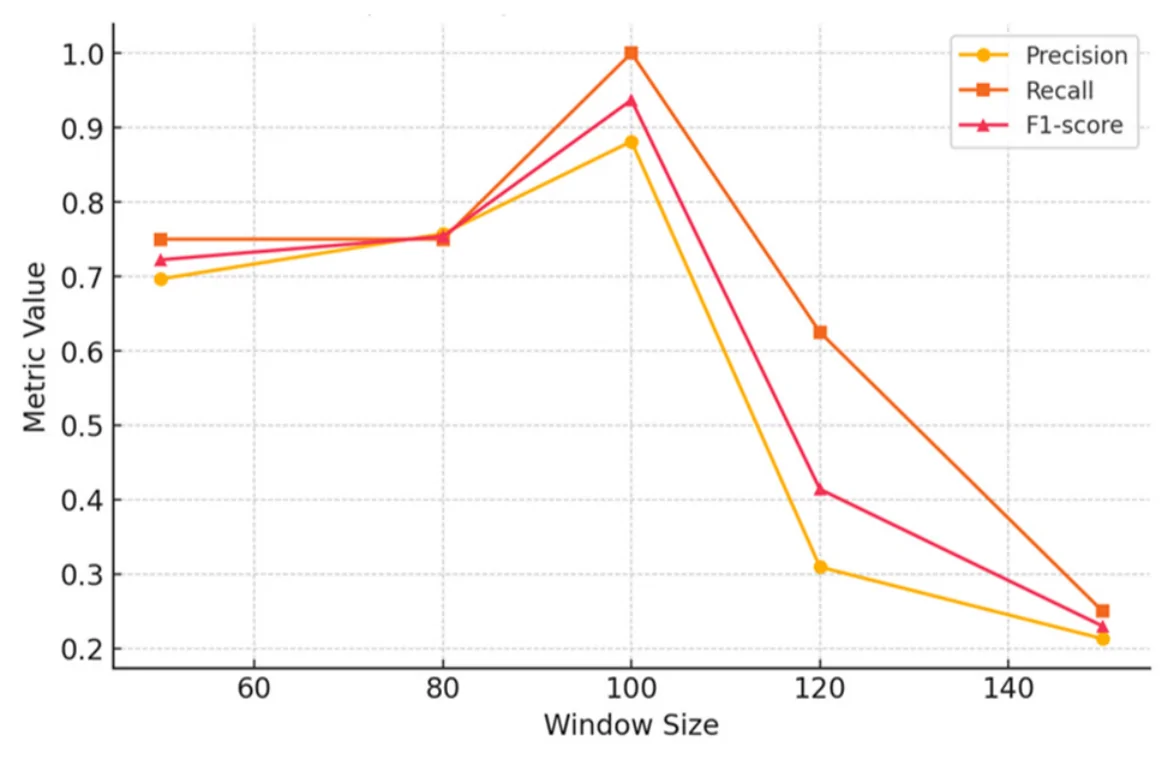
Impact of detection window length on Precision, Recall, and F1-score.

**Figure 6:**
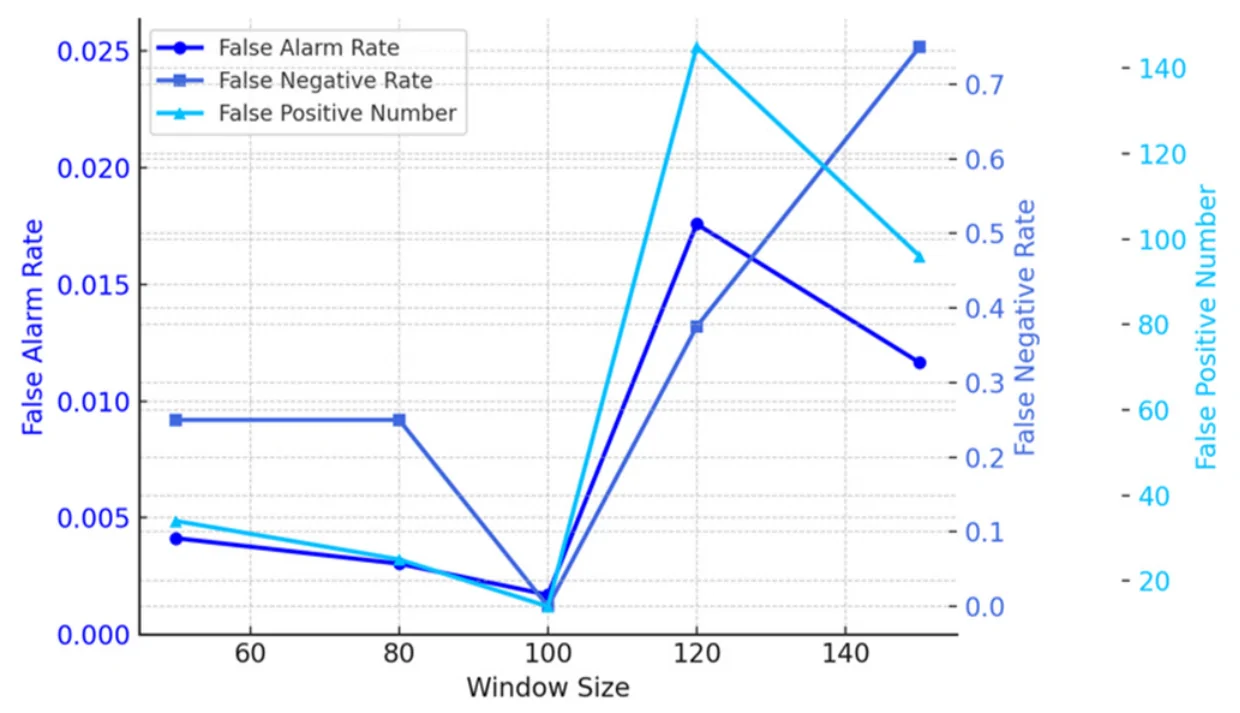
Impact of detection window length on FAR, FNR, and FP.

For detection window length, five settings were tested: 50, 80, 100, 120, and 150 time steps. The effects on positive indicators and negative indicators are shown in Figure 5 and Figure 6, respectively.

The results indicate that an excessively short window provides insufficient temporal context for capturing methane migration, ventilation modulation, and multi-sensor coupling. In contrast, an overly long window introduces redundant historical fluctuations and may weaken the model’s sensitivity to local abnormal changes. The best overall performance is obtained when the detection window length is 100, which provides a suitable balance between temporal-context representation and abnormal-response localization.

**Figure 7:**
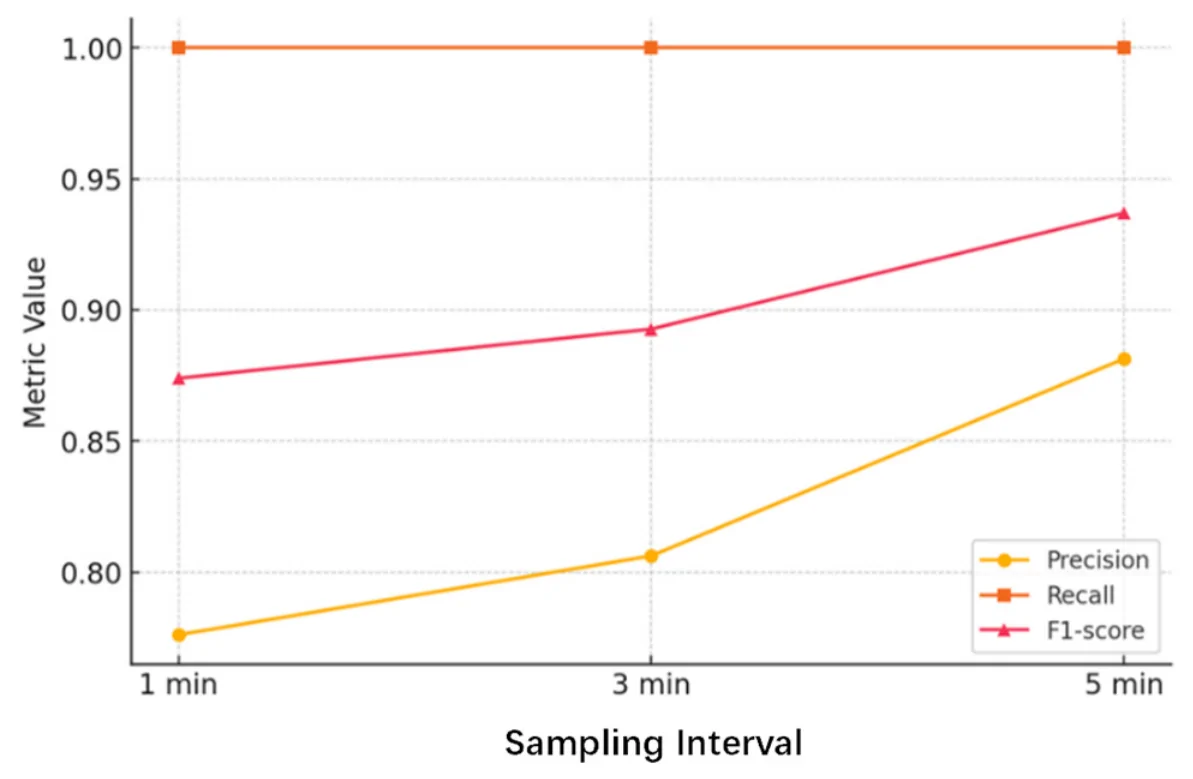
Impact of sampling interval on Precision, Recall, and F1-score.

**Figure 8:**
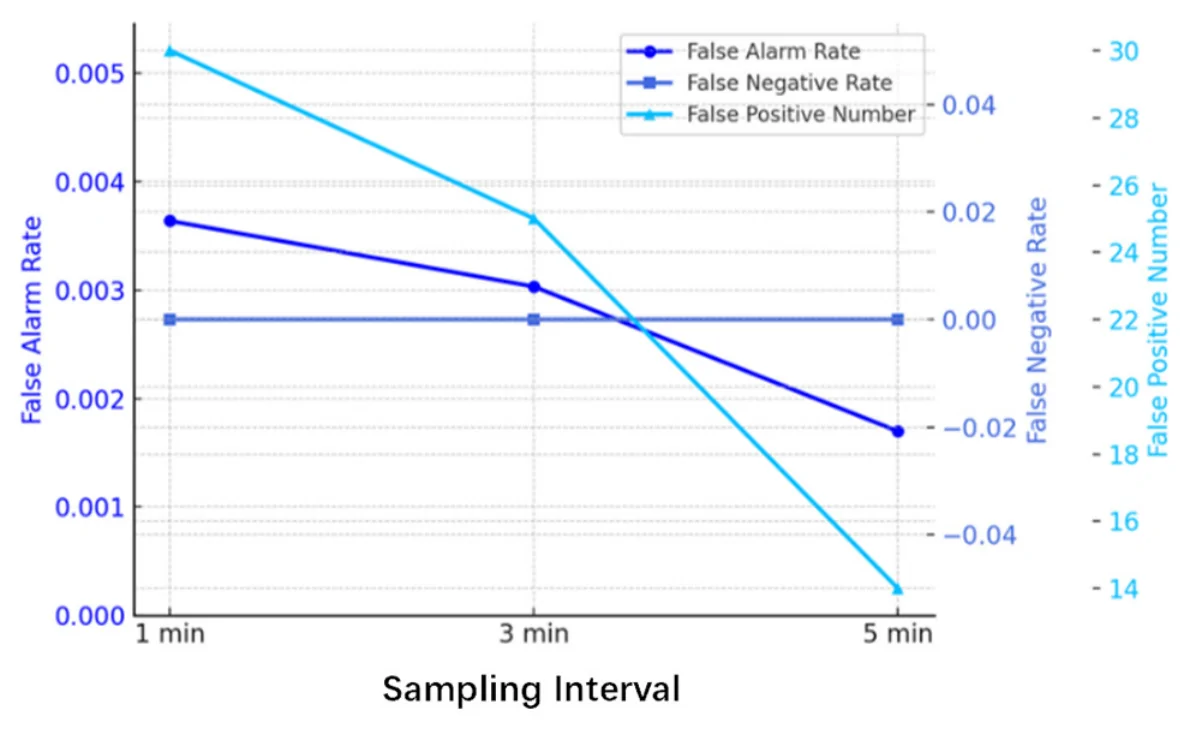
Impact of sampling interval on FAR, FNR, and FP.

For the sampling interval, three aggregated sampling intervals were compared: 1 min, 3 min, and 5 min. The 1, 3, and 5 min datasets were generated from the common 15 s aligned sequence to evaluate the influence of temporal resolution. Based on this sensitivity analysis, the 5 min configuration was selected for the main comparative and ablation experiments because it provided the best balance between abnormal-event coverage and false-alarm suppression in the present dataset. These datasets were generated by further aggregating the 15 s aligned sensor sequence described in Section 3.2. The corresponding results are shown in Figure 7 and Figure 8.

A shorter sampling interval preserves more transient details, but it may also retain short-term noise and increase the probability of false alarms. A longer sampling interval smooths random fluctuations and reduces false positives, but it may weaken the model’s ability to capture rapidly evolving abnormal signals. In this experiment, the 5 min aggregated sequence achieves a better balance by maintaining high Recall while reducing FAR and FP. Nevertheless, this result should not be interpreted as a universal preference for lower temporal resolution. In practical deployment, the sampling interval should be selected according to the required warning timeliness, sensor noise level, data transmission conditions, and acceptable false-alarm rate.

**Figure 9:**
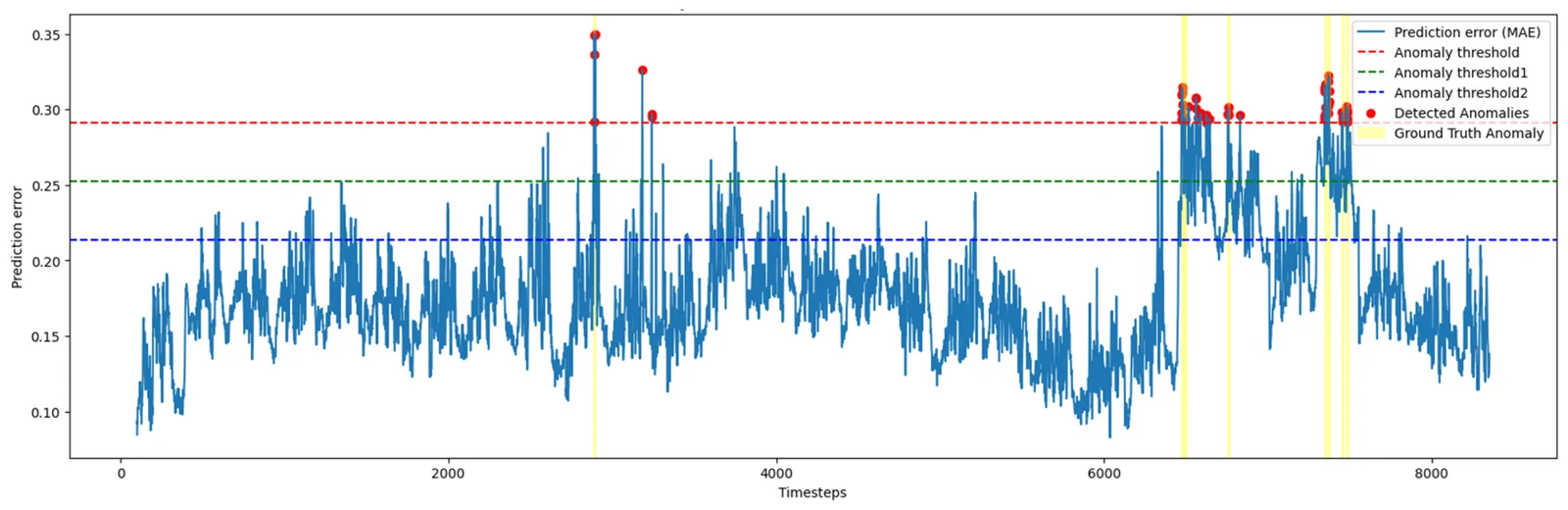
Impact of anomaly threshold on detection results.

For the anomaly threshold, different standard-deviation multiples were used to set the anomaly-decision boundary, as shown in Figure 9.

When the threshold is set too low, mild reconstruction-error fluctuations may frequently exceed the decision boundary, resulting in excessive false alarms. When the threshold is too high, weak precursor-related abnormal intervals may be missed. As the threshold decreases, more mild reconstruction-error fluctuations are classified as abnormal, increasing false alarms during normal production. Conversely, increasing the threshold suppresses these weak fluctuations but may also increase the risk of missing less pronounced abnormal responses. Among the three candidate settings evaluated in this study, μ+σ, μ+2σ, and μ+3σ, the μ+3σ threshold produced the most favorable trade-off for the present dataset, substantially reducing false alarms while retaining the detection of the annotated abnormal intervals.

Accordingly, μ+3σ was adopted for the main experiments. This result should not be interpreted as demonstrating that the three-standard-deviation rule is universally optimal for underground mine monitoring. The appropriate threshold may vary with sensor noise, operating conditions, mine-specific anomaly prevalence, and the relative costs of false alarms and missed detections. More adaptive thresholding approaches, such as percentile-based, extreme-value-based, or online adaptive strategies, should be investigated in future multi-mine validation studies.

### 3.7. Computational Complexity and Deployment Considerations

The computational cost of GasNet mainly arises from the GCN, TimesNet, and cross-attention modules. Let *N* denote the number of sensor nodes, *T* the input sequence length, $\left|E\right|$ the number of graph edges, and d the latent feature dimension. For a sparse engineering-prior graph, the graph-convolution operation has an approximate complexity of $O\left(T\left|E\right|d\right)$, which scales with the number of physically retained sensor relationships rather than N2. TimesNet additionally performs frequency-domain period discovery using the fast Fourier transform, with an FFT-related cost of approximately $O\left(NT\log T\right)$, followed by multi-period temporal feature extraction. The cross-attention fusion introduces an additional sequence-interaction cost that scales approximately with $O\left(T^{2}d\right)$ for the adopted attention formulation. Consequently, the overall computational requirement is dominated by temporal–spectral modeling and cross-attention rather than by the sparse GCN branch.

An important practical feature is that the engineering-prior graph is sparse and contains only 16 monitoring nodes in the present case. Therefore, graph message passing introduces limited additional computational overhead compared with the temporal backbone. Replacing the prior graph with a fully connected graph increases the number of message-passing edges but does not improve detection performance, as demonstrated by the ablation analysis. Thus, the prior graph provides both a physically constrained representation and a relatively efficient spatial information pathway.

In the main experiments, the sampling interval is 5 min and the input window contains 100 time steps, corresponding to an 8 h 20 min historical context. This context length should not be interpreted as computational or warning latency. With a one-step sliding update, a new anomaly score can be generated whenever a new 5 min monitoring sample becomes available. Therefore, the nominal decision-update interval is 5 min, while neural-network inference itself is performed only after the current window has been formed.

The original experiments were performed on an NVIDIA RTX 3090 GPU with 24 GB memory. Exact per-sample inference latency, peak GPU-memory allocation, serialized model size, and parameter-count profiling were not retained in the archived experimental records. Therefore, estimated profiling values are not retrospectively reported as measured results. From a deployment perspective, the 5 min data-update cycle provides a relatively relaxed computational interval for online inference; nevertheless, standardized profiling on the target hardware is still required before claiming real-time deployability.

The baseline models also differ substantially in their theoretical computational characteristics. Transformer and Informer are attention-based sequence models, with Informer specifically designed to reduce the computational burden of long-sequence attention. Autoformer and FEDformer introduce decomposition and frequency-domain operations, while Crossformer additionally models cross-variable dependencies. DLinear has the lowest structural complexity among the compared models because it relies primarily on linear decomposition and mapping. FiLM performs frequency-domain sequence modeling. GasNet introduces an additional sparse GCN branch and cross-attention fusion on top of temporal–spectral modeling; therefore, its architectural complexity is higher than that of lightweight models such as DLinear but remains compatible with the relatively low-dimensional 16-sensor input used in the present application.

## 4. Interpretability and Engineering Tracing

### 4.1. Macroscopic Interpretation via Cross-Attention

**Figure 10:**
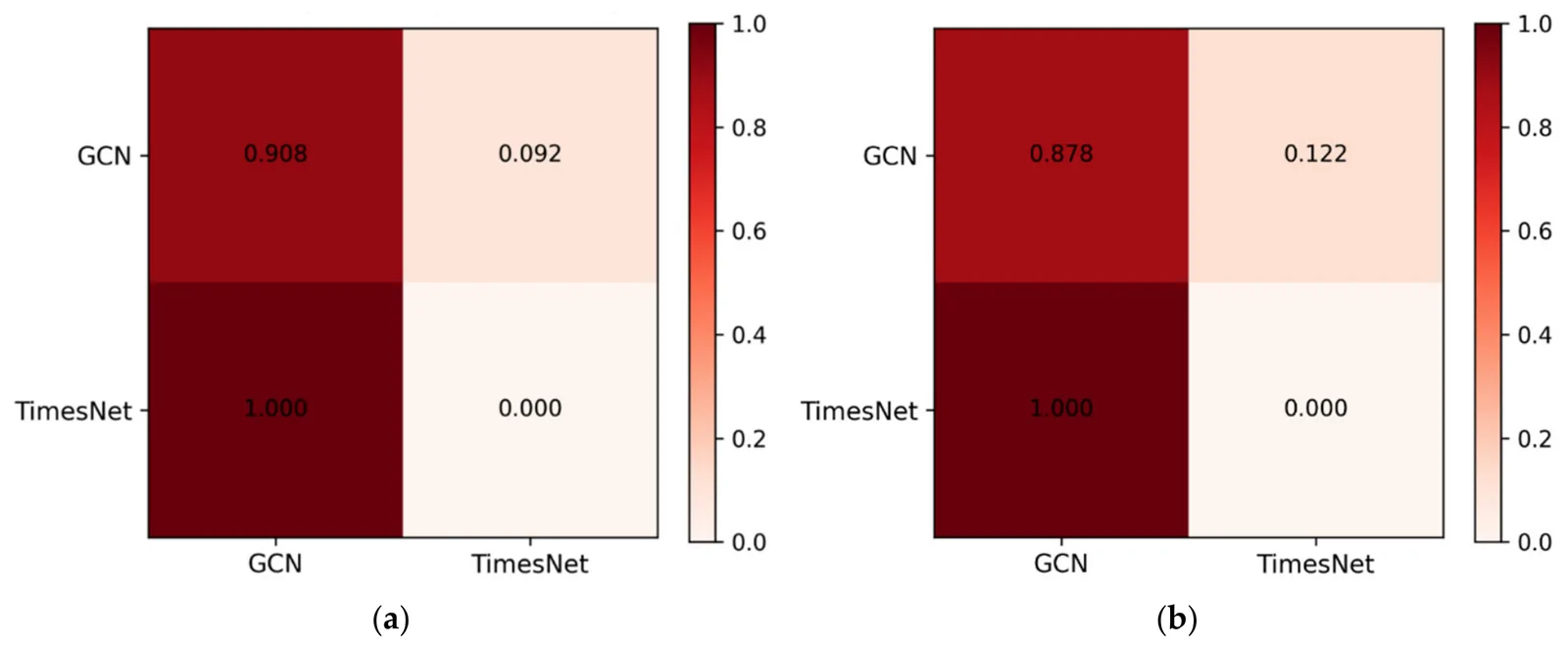
Comparison of cross-attention weight distributions in GasNet: (**a**) abnormal samples; (**b**) normal samples.

The cross-attention module provides a macroscopic view of how GasNet integrates spatial–topological features from the GCN branch and temporal–spectral features from the TimesNet branch. To examine whether the feature-fusion behavior changes under different monitoring states, the attention weights were statistically compared between normal intervals and precursor-related abnormal intervals. The results are shown in Figure 10.

The results show that the TimesNet branch maintains a strong dependence on GCN-related spatial–topological features in both normal and abnormal states. Specifically, the attention weight from TimesNet to GCN remains close to 1.000, indicating that temporal–spectral pattern extraction is consistently influenced by the sensor-topological representation. This result is reasonable because methane-related monitoring signals in an underground working face are not isolated temporal fluctuations; they are jointly affected by ventilation pathways, sensor locations, and equipment-induced disturbance propagation.

In contrast, the dependence of the GCN branch on TimesNet-related features decreases during abnormal intervals. The GCN-to-TimesNet attention weight decreases from 0.122 under normal conditions to 0.092 under abnormal conditions, representing a reduction of approximately 24.6%. Meanwhile, the self-dependence of the GCN branch increases from 0.878 to 0.908. From the model perspective, this change suggests that GasNet relies more strongly on spatial–topological sensor relationships when identifying precursor-related abnormal intervals. In other words, abnormal intervals are more likely to be supported by coordinated multi-sensor responses constrained by the engineering-prior graph, rather than by isolated temporal fluctuations at a single sensor.

From an engineering viewpoint, this finding is consistent with the monitoring characteristics of underground gas-related abnormal states. Under normal production, methane fluctuations can often be explained by periodic cutting, airflow adjustment, or short-term operational disturbance, so temporal–spectral patterns remain informative. During precursor-related abnormal intervals, however, gas-related signals may show more coordinated changes along the ventilation route and sensor topology, making spatial–topological information more prominent in the model decision process. The increased GCN self-dependence therefore suggests that GasNet captures a transition from ordinary time-series fluctuation to spatially linked abnormal sensor response.

It should be emphasized that the cross-attention weights characterize the internal feature-fusion behavior of GasNet rather than providing direct causal attribution of individual sensor variables. Accordingly, the observed changes in GCN-to-TimesNet and TimesNet-to-GCN dependence are interpreted as macroscopic model evidence and are not considered in isolation. Instead, they are examined together with the ablation results in Section 3.5, where removing the graph-related structure or engineering-prior topology substantially degraded abnormal-interval detection. The consistency between these analyses supports the functional relevance of the observed attention shifts and indicates that GasNet evaluates abnormal intervals through the joint representation of spatial–topological dependence and temporal–spectral evolution rather than through isolated single-sensor threshold exceedance or temporal variation alone. Nevertheless, formal perturbation-based faithfulness testing of the attention weights was not performed in the present study.

### 4.2. Sensor-Time Interpretation via Knowledge Distillation

Macroscopic cross-attention analysis reveals how GasNet balances spatial–topological and temporal–spectral information, but it cannot directly identify the sensor channels and temporal segments that contribute strongly to a specific warning. Therefore, the student model is not regarded as a faithful functional replica of GasNet. Because direct sample-wise teacher–student fidelity measures were not retained in the original experiment and the student shows substantially lower predictive performance, its activation maps are interpreted only as heuristic surrogate sensor-time evidence associated with the teacher-informed distillation process, rather than as reliable attribution of individual GasNet decisions. Accordingly, the activation maps are used only for exploratory warning review and should be considered together with GasNet cross-attention behavior, ablation results, and field monitoring information.

**Figure 11:**
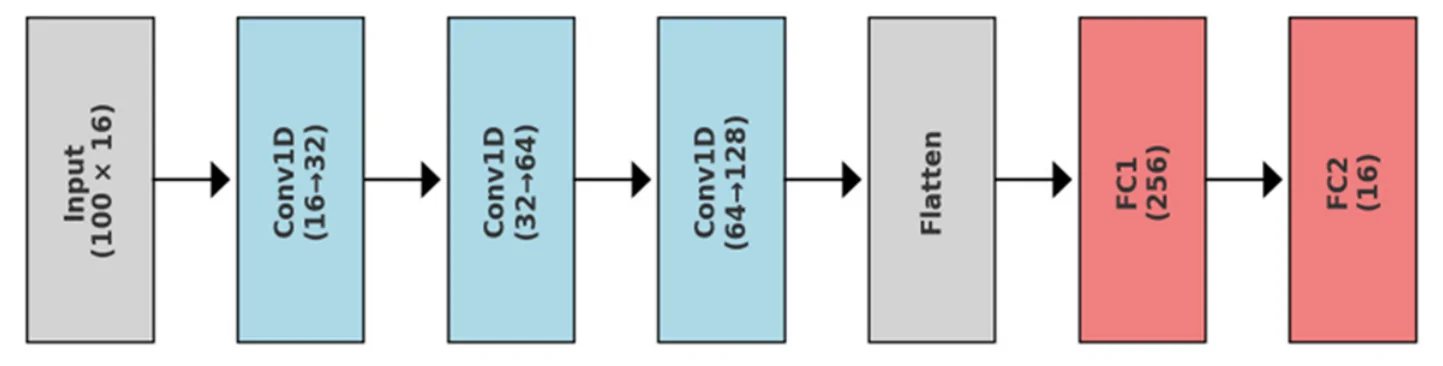
Architecture of the 1D-CNN student model for knowledge-distillation-based interpretation.

**Figure 12:**
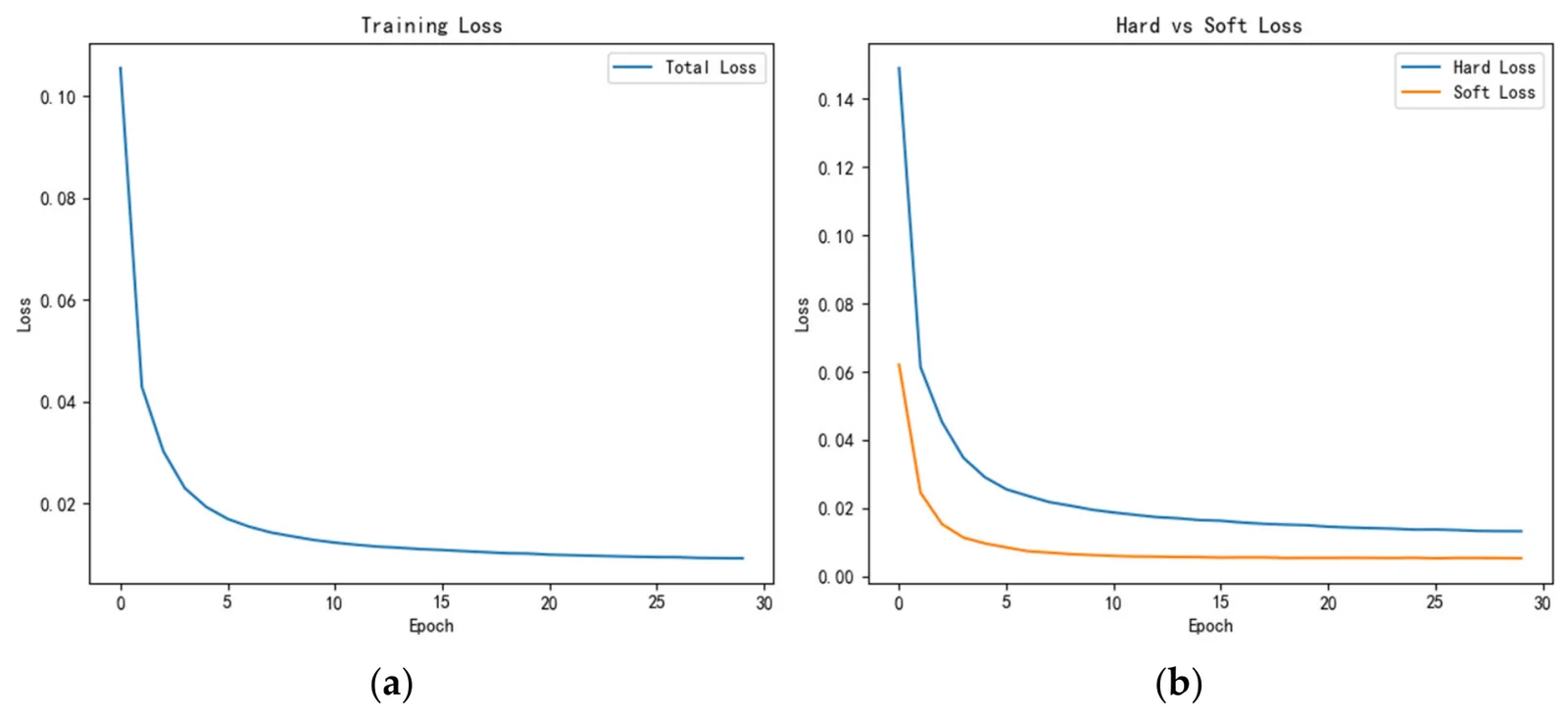
Distillation training process of the student model: (**a**) total loss curve; (**b**) hard-label and soft-target loss curves.

The distillation procedure was conducted entirely using the training set. GasNet was first trained using the predominantly normal training data, after which its parameters were fixed. The same training-set samples were subsequently used to train the 1D-CNN student. Consistent with the training assumption used for GasNet, all training samples were assigned the normal hard target (y = 0); no manually annotated abnormal labels were available or introduced during student training. Meanwhile, the fixed GasNet teacher generated continuous soft outputs for the same training samples, which were used as soft targets to transfer the teacher’s learned response behavior to the student. The student was therefore optimized jointly by the normal-state hard-target loss and the teacher-derived soft-target loss described in Section 2.5. The annotated abnormal intervals in the test period were not used during student training. After completion of distillation, both teacher and student models were applied to the test set for evaluation, and the trained student was further used for sensor-time activation analysis. The architecture of the distilled 1D-CNN student model is shown in Figure 11, and the corresponding distillation training process is presented in Figure 12.

To quantitatively examine the fidelity of the distilled model, the teacher and student were evaluated on the same test set. GasNet achieved a Precision of 0.8814, a Recall of 1.0000, an F1-score of approximately 0.937, an FAR of 0.0017, an FNR of 0, and 14 false positives. The 1D-CNN student achieved a Precision of 0.5353, a Recall of 0.8750, an F1-score of 0.6635, an FAR of 0.0096, an FNR of 0.1250, and 79 false positives. These results show that the student retains relatively high sensitivity to the abnormal intervals but does not fully reproduce the decision boundary of the GasNet teacher. In particular, the increased number of false positives indicates that model simplification leads to a loss of discrimination capability.

Given the substantial predictive discrepancy between the student and teacher models, the student activation maps should be interpreted only as heuristic surrogate evidence of sensor-time patterns associated with the teacher-informed distillation process, rather than as reliable attribution of individual GasNet decisions. This distinction is important when interpreting the subsequent activation results: sensor channels and temporal segments highlighted by the student provide complementary evidence for warning tracing but should not be regarded as exact attribution values for every GasNet prediction.

**Figure 13:**
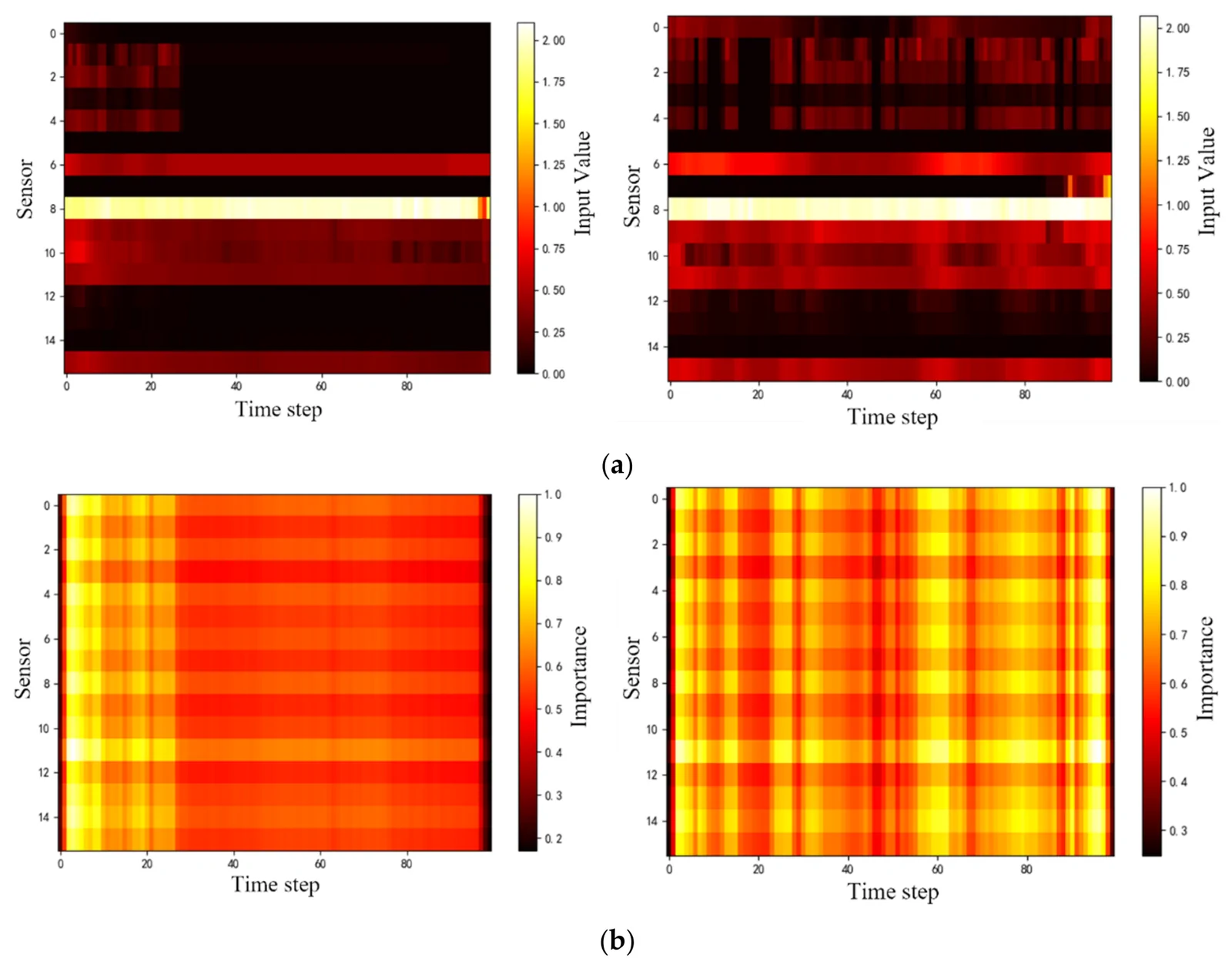

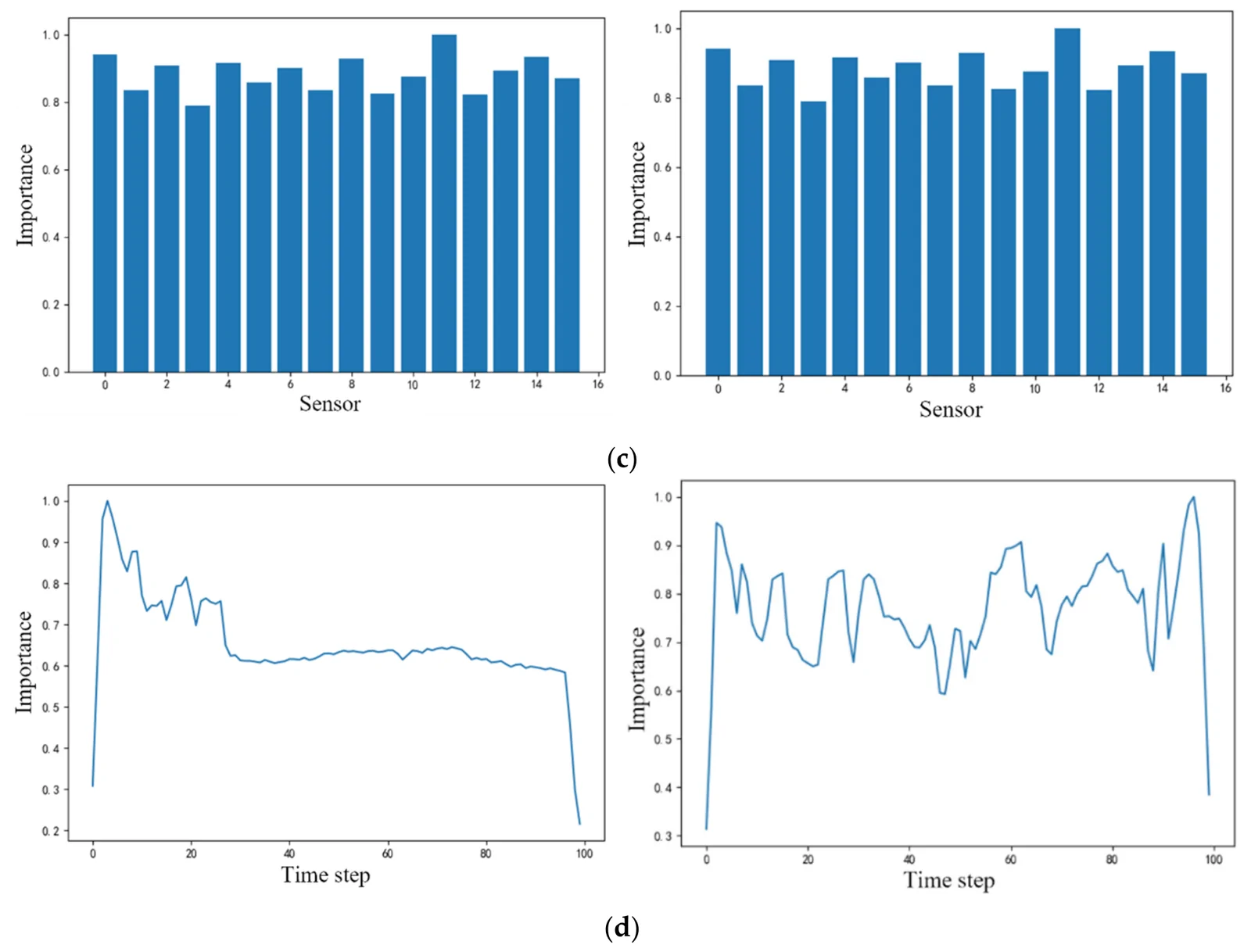
Global sensor-time interpretation results of the distilled 1D-CNN student model: (**a**) input heatmaps of normal and abnormal samples; (**b**) feature-importance heatmaps; (**c**) sensor-channel importance comparison; and (**d**) time-step importance comparison.

After distillation, activation mapping was applied to the last convolutional layer of the student model to analyze sensor-time contributions. Figure 13 summarizes the global interpretation results for normal and abnormal samples. The sensor channels were arranged in the following order: JZ, cmjsd, zbp, zjg, ybp, yjg, wz, FC, FS, T0, T1, T2, T30, T31, T32, and T5. As shown in Figure 13a, abnormal samples exhibit more fragmented and pulse-like variations across multiple channels than normal samples. Figure 13b further shows that the feature-importance distribution under abnormal conditions becomes more localized, with several high-response regions concentrated in specific sensor-time areas. This indicates that the student model does not treat all variables and time steps equally but selectively amplifies monitoring segments that deviate from normal coupling patterns.

The sensor-channel importance comparison in Figure 13c shows that methane-related channels, especially the return-airway methane sensor, T2, maintain high importance in both normal and abnormal samples. This is reasonable because T2 reflects the integrated gas response transported by the return airflow and is sensitive to changes in overall gas emission and ventilation stability. Meanwhile, the activation of wind velocity and shearer-related variables provides contextual evidence for distinguishing normal production-induced fluctuations from precursor-related abnormal deviations. Figure 13d shows that abnormal samples have more pronounced local peaks in the time-step importance curve, suggesting that the warning output is mainly supported by several critical temporal segments rather than by the entire input window uniformly.

**Figure 14:**
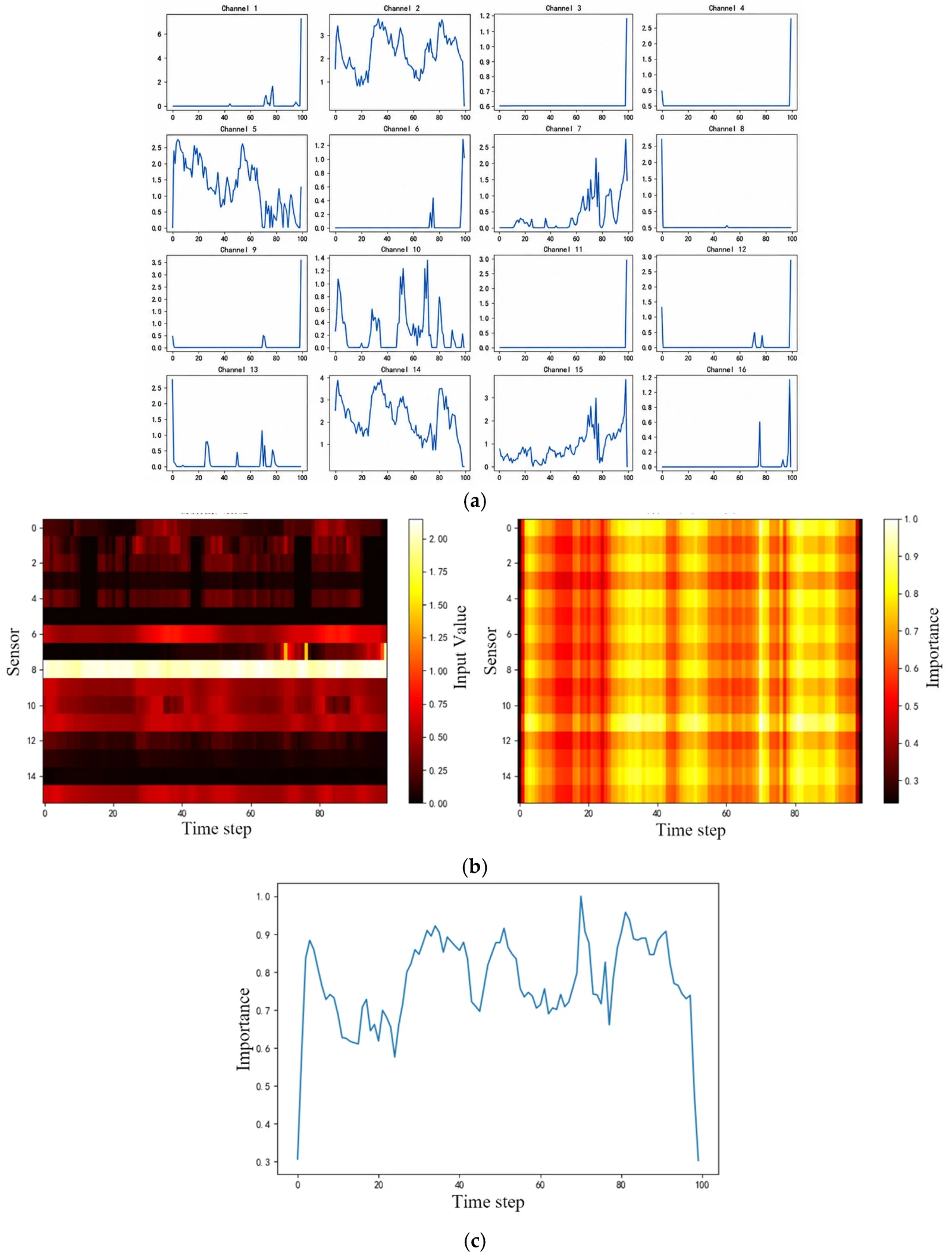
Case-level sensor-time interpretation results for the abnormal interval on 25 April 2022: (**a**) channel activation feature map; (**b**) input and feature-importance heatmaps; and (**c**) time-step importance curve.

To further illustrate the microscopic interpretation process, the abnormal interval on 25 April 2022 was selected as a representative case. Field records indicate that this interval involved wind-velocity fluctuation and abnormal deviations in several monitoring variables, including T0, T5, and FS. During this period, production-operation intensity increased and temporary ventilation disturbance occurred, resulting in multi-sensor fluctuations and a potential abnormal monitoring state. The corresponding case-level interpretation results are shown in Figure 14.

As shown in Figure 14a, the student model produces stronger activation responses in several sensor channels during this abnormal interval, indicating that the warning is supported by localized multi-channel evidence. Figure 14b shows that the high-importance regions are mainly concentrated around methane-related channels and wind velocity, suggesting that the warning is not triggered by a single isolated sensor spike, but by the combined variation in gas concentration and ventilation modulation. Figure 14c further shows that several filters exhibit a clear increase in importance around the 80th to 100th time steps, indicating that the later part of the input window contributes more strongly to the final warning output. This temporal localization is consistent with the field observation that multi-sensor fluctuations became more evident in the later stage of the abnormal interval.

Overall, the microscopic tracing results provide sensor- and temporal-level evidence that complements the macroscopic cross-attention analysis. Methane-related channels contribute strongly to the distilled warning representation, while wind-velocity and shearer-related variables provide additional modulation context. However, because the student model does not closely reproduce the predictive behavior of GasNet, these activation patterns are used only as heuristic surrogate evidence for exploratory warning review and should not be interpreted as faithful attribution of the teacher model. They should therefore be considered together with the GasNet attention analysis, module-ablation results, and field-operation records during engineering review.

### 4.3. Implications for On-Site Warning Review

The interpretability results in Section 4.1 and Section 4.2 provide complementary evidence for on-site warning review. Cross-attention analysis explains the macroscopic information source of a warning, while knowledge-distillation-based sensor-time interpretation identifies the key sensor channels and temporal segments that contribute to the warning output. Together, these two levels of interpretation can support a practical review process for precursor-related anomaly detection in underground mine sensor networks.

When the cross-attention results show increased dependence on the GCN branch, engineers should first check whether methane-related signals exhibit spatially linked changes along the ventilation route. For example, simultaneous or sequential changes in T0, T1, T5, and T2 may indicate that the warning is not caused by an isolated sensor fluctuation, but by a coordinated gas-response pattern. In this case, the warning should be reviewed together with ventilation conditions, return-airway methane concentration, upper-corner gas accumulation, and local gas drainage records.

When sensor-time interpretation shows high activation in methane-related channels, the warning should be interpreted as being mainly supported by core gas-response evidence. If the high-importance channels are concentrated around T0, T5, or T2, the review should focus on upper-corner gas accumulation, return-airway methane variation, and whether abnormal gas emission is consistent with the current mining position. If high activation also appears in FS or shearer-operation variables, engineers should further examine whether the methane fluctuation is associated with airflow disturbance, cutting intensity, shearer position, or short-term operational changes.

This interpretation logic can help distinguish different warning scenarios. If the warning is characterized by coordinated methane responses across multiple sensors, increased dependence on the engineering-prior graph, and temporally concentrated activation, it should be treated as a high-priority abnormal interval requiring field verification. If the warning is dominated by a single short-duration sensor fluctuation and is strongly synchronized with ordinary shearer operation or ventilation adjustment, it may represent a lower-confidence alarm that requires manual confirmation before strong intervention measures are taken.

Therefore, GasNet should be regarded as a decision-support tool rather than an automatic replacement for engineering judgment. Its warning output identifies monitoring intervals that deviate from learned normal production patterns, while the interpretability module provides traceable model evidence for review. In practical deployment, the model output should be combined with gas drainage records, drilling feedback, ventilation adjustment logs, geological exposure, and production–operation information. This forms a closed-loop process of model warning, interpretable evidence tracing, engineering verification, and on-site response, which can improve the reliability and usability of dynamic abnormal-state monitoring in underground coal mines.

## 5. Discussion

The results demonstrate that integrating engineering structure priors with graph–temporal–spectral feature learning can improve precursor-related anomaly detection in underground mine sensor networks. Compared with purely time-series models, GasNet achieves higher Recall and fewer false alarms because it does not treat multi-source monitoring variables as independent or homogeneous sequences. Instead, the engineering-prior sensor graph constrains feature aggregation according to ventilation pathways, sensor deployment, and shearer-related coupling relationships. This differs from graph-based approaches in which connectivity is learned mainly from statistical dependencies or used primarily for forecasting. In GasNet, the graph acts as an explicit engineering constraint on spatial message passing within a reconstruction-based anomaly-detection framework. This design is particularly important in underground working faces, where methane responses are jointly affected by gas emission, airflow dilution, coal fragmentation, mining disturbance, and local geological conditions.

The proposed framework also highlights the importance of sensor-role-aware modeling. Methane-related sensors provide direct gas-response information, while airflow, dust, and shearer-operation sensors provide environmental and operational context. If all variables are treated equally during anomaly scoring, normal production-induced fluctuations in auxiliary sensors may lead to false alarms. By decoupling the global reconstruction objective from the sensor-role-aware anomaly score, GasNet learns the coupling patterns among all sensor variables during training while emphasizing methane-related reconstruction deviations during inference. This strategy improves the model’s ability to distinguish precursor-related abnormal intervals from routine production disturbances.

The interpretability results further show that GasNet provides more than a binary warning output. Cross-attention analysis reveals the macroscopic feature-fusion behavior between spatial–topological and temporal–spectral representations, while knowledge-distillation-based activation mapping identifies key sensor channels and high-contribution time segments. This two-level interpretation is valuable for on-site warning review because field engineers need to understand whether an alarm is mainly supported by coordinated methane responses, ventilation disturbance, shearer-operation changes, or localized temporal fluctuations. Therefore, GasNet can support engineering verification and decision-making rather than only providing an isolated anomaly score.

Given that the current validation is based on a single working face and only eight annotated abnormal intervals, the present results should be regarded as a field proof of concept rather than evidence of established cross-mine generalizability. However, several limitations should be noted.

1. The validation scope remains limited. The current study is based on data from a single working face. Although the 31002 working face has complex gas-geological conditions and provides a realistic field scenario, the generalization ability of GasNet still needs to be verified across different mines, ventilation modes, sensor layouts, and geological conditions. Future studies should conduct cross-working-face and cross-mine validation to evaluate the robustness of the proposed framework under different production environments. In addition, the comparative experiments were based on a single final training run for each model, and repeated-run variability was not quantified. Therefore, the reported differences, particularly the relatively small F1-score gap between GasNet and Informer, should be interpreted as descriptive rather than statistically conclusive. Future work should report repeated-run means ± standard deviations, confidence intervals, and paired statistical comparisons using larger multi-mine event sets. The current benchmark primarily covers representative temporal-modeling architectures together with graph-based ablations of GasNet. Dedicated anomaly-detection architectures based on graph attention, variational modeling, adversarial learning, and deep reconstruction were not exhaustively evaluated. Future multi-mine studies should therefore include a broader standardized anomaly-detection benchmark. In addition, the point-adjustment procedure uses ground-truth interval boundaries during offline evaluation and may favor continuity-oriented interval detection; therefore, Recall and F1-score should not be interpreted as raw online point-wise performance.
2. The ground-truth definition remains subject to field-record uncertainty. The abnormal labels used in this study are precursor-related abnormal intervals rather than actual coal and gas outburst accident labels. The event occurrence times were obtained from contemporaneous abnormal-event records provided by mine technical personnel, after which a predefined 1 h pre-event rule was used to generate the corresponding evaluation intervals. The intervals were not independently re-annotated by multiple experts, and therefore inter-rater agreement could not be quantified. In addition, the retained records do not allow a reliable one-to-one reconstruction of the specific engineering subtype associated with every event. These factors introduce uncertainty into the ground-truth definition. Future studies should employ standardized event documentation, independent multi-expert review, and longer-term monitoring records to improve label reliability.
3. The engineering-prior graph requires dynamic maintenance and further robustness evaluation. Practical deployment should therefore include graph updating, sensor-status checking, and topology-consistency verification mechanisms. Although the current ablation study evaluates a substantial topology perturbation by replacing the engineering-prior graph with a fully connected graph, it does not exhaustively examine intermediate graph-density levels, structured edge removal, or uncertainty in individual engineering edges. Future studies should investigate random and mechanism-specific edge perturbations to quantify topology robustness under changes in sensor layout, ventilation conditions, and engineering assumptions.
4. The interpretability evidence remains approximate rather than causal. Because the student model shows substantial predictive disagreement with GasNet, its activation maps cannot be regarded as faithful local explanations of individual teacher predictions. The cross-attention weights characterize the internal fusion behavior between spatial–topological and temporal–spectral representations, while the distilled 1D-CNN provides localized sensor-time evidence. Although the student model retains relatively high abnormal-interval sensitivity, its predictive performance does not fully match that of the GasNet teacher. In addition, formal perturbation-based faithfulness metrics, deletion/insertion tests, and independent comparisons with attribution methods such as SHAP or Integrated Gradients were not performed. Therefore, the current explanations should be interpreted as complementary model evidence rather than causal attribution. Future work should quantitatively evaluate explanation faithfulness using multiple attribution and perturbation-based methods. In addition, direct sample-wise fidelity measures between the GasNet teacher and the distilled student were not retained. Given the substantial predictive discrepancy between the two models, the student activation maps cannot be regarded as faithful local explanations of individual GasNet predictions and are therefore interpreted only as heuristic surrogate evidence.
5. Real-time deployment still requires further engineering validation. Although the 5 min monitoring interval provides a practical decision-update timescale, exact inference latency, peak memory consumption, model-size constraints, data-transmission delay, missing sensor streams, and alarm-response procedures should be systematically evaluated on the target industrial hardware before real-time deployment. In particular, edge-device profiling, model compression, sensor-failure handling, and integration with existing mine safety monitoring platforms should be further investigated before GasNet is used in routine production environments.

From the perspective of trustworthy AI deployment, the design of GasNet also has implications for transparency, human oversight, and risk management in safety-critical monitoring. Although the present study does not establish regulatory compliance or determine whether a future deployment would fall within a specific legal classification, several principles in current AI governance frameworks are relevant to its engineering use. Article 13 of the EU Artificial Intelligence Act emphasizes that high-risk AI systems should provide sufficient transparency for deployers to interpret system outputs and use them appropriately, together with information on intended purpose, performance limitations, robustness, interpretation of outputs, human oversight, and maintenance requirements [26]. The NIST AI Risk Management Framework similarly emphasizes lifecycle-oriented governance through the Govern, Map, Measure, and Manage functions, including documentation, risk measurement, monitoring, and clearly assigned human responsibilities [27]. IEEE 7001-2021 further emphasizes measurable and testable transparency [28], while IEEE 7000-2021 provides a process-oriented framework for incorporating ethical and risk-related considerations into system design [29].

These principles are consistent with the intended role of GasNet as a decision-support tool rather than an autonomous replacement for engineering judgment. In practical deployment, anomaly scores should therefore be accompanied by interpretable sensor-topology and sensor-time evidence, documented model limitations and operating conditions, sensor-status and graph-consistency monitoring, and procedures that allow trained personnel to review, override, or disregard model warnings when inconsistent with field evidence. Model performance, data quality, graph validity, and warning behavior should also be periodically reviewed after deployment. Such measures would improve transparency and traceability while reducing the risk of inappropriate reliance on automated warning outputs.

## 6. Conclusions

This study proposed GasNet, an interpretable graph–temporal–spectral fusion framework for precursor-related anomaly detection in underground mine sensor networks. By integrating multi-source sensor-role organization, engineering-prior graph construction, reconstruction-based anomaly detection, and multi-level interpretability, the proposed framework provides a sensor-fusion-based solution for dynamic abnormal-state monitoring in underground coal mines. The main conclusions are as follows:

1. A hierarchical sensor-role organization was established by distinguishing methane-related core sensors from environmental and operational modulation sensors. This design enables the model to analyze methane responses together with airflow, dust, and shearer-operation contexts, providing a physically meaningful basis for identifying precursor-related abnormal intervals from multi-source monitoring data.
2. An engineering-prior sensor graph was constructed using ventilation pathways, sensor deployment, and equipment-coupling relationships. Combined with GCN, TimesNet, and cross-attention, GasNet can jointly capture spatial–topological dependencies and temporal–spectral patterns in underground mine sensor sequences. Field validation on the investigated 31002 fully mechanized working face showed that GasNet achieved a Precision of 0.881, a Recall of 1.000, an F1-score of 0.937, an FAR of 0.0017, and zero missed detections, with the highest numerical F1-score among the seven representative time-series baselines in the present dataset.
3. Ablation experiments confirmed the necessity of the main components. GCN-only was sensitive to abnormal intervals but produced excessive false alarms, while TimesNet-only missed several abnormal intervals. Removing the engineering-prior adjacency matrix substantially reduced the F1-score and increased the missed-warning risk. These results demonstrate that engineering-prior topology, spatial modeling, temporal–spectral modeling, and cross-attention fusion jointly contribute to the improved detection performance of GasNet.
4. The proposed multi-level interpretability framework provided traceable evidence for warning review. Cross-attention analysis revealed the macroscopic fusion behavior between spatial–topological and temporal–spectral features, while knowledge-distillation-based activation mapping identified key sensor channels and high-contribution time segments. These explanations can support on-site engineering verification, but they should be interpreted as model evidence rather than direct physical source inversion.

Overall, the present field study demonstrates the feasibility of GasNet as an interpretable multi-source sensor-fusion framework for detecting abnormal monitoring intervals that deviate from learned normal production patterns in the investigated working face. Future work will focus on multi-working-face and multi-mine validation, systematic assessment of model robustness and interpretability, and practical deployment under different operating conditions. For industrial implementation, the engineering-prior graph should be dynamically updated when sensor failures, missing data streams, sensor relocation, or ventilation-layout changes occur, while model outputs should be integrated with sensor-status monitoring and engineering verification mechanisms.

## Figures and Tables

**Table 1:** Multi-source sensor variables and hierarchical monitoring roles for underground mine abnormal-state detection.

Monitoring Role	Sensor Type	Sensor Code	Physical Quantity	Role in GasNet
Core sensor	Upper-corner methane	T0	Methane concentration	Reflects gas accumulation near the upper corner and goaf-side region.
	Working-face methane	T1		Captures gas emission response during cutting and face advancement.
	Return-airway methane	T2, T5		Represents integrated gas outflow and regional gas-response stability.
	Intake-airflow methane	T30, T31, T32		Provides upstream background methane information.
	Onboard shearer methane	JZ		Captures methane response near the moving cutting source.
Environmental modulation	Wind velocity	FS	Airflow velocity	Describes ventilation dilution and airflow disturbance.
	Dust concentration	FC	Dust concentration	Reflects coal fragmentation and cutting-induced particulate response.
Operational modulation	Shearer traction speed	cmjsd	Speed	Represents mining-operation intensity.
	Shearer motor/current variables	zbp, zjg, ybp, yjg	Current/frequency-conversion output	Describes cutting load and equipment disturbance.
	Shearer position	wz	Position	Provides spatial context of the mining disturbance source.

Note: The specific number of sensors (e.g., intake sensors T30–T32) may vary depending on the ventilation mode (W-type), but the hierarchical roles remain consistent.

**Table 2:** Adjacency matrix representation of the engineering-prior sensor graph.

	jz	cmjsd	zbp	zjg	ybp	yjg	wz	FC	FS	T0	T1	T2	T30	T31	T32	T5
jz	1	0	0	0	0	0	0	0	0	1	0	0	0	0	0	0
cmjsd	1	1	1	1	1	1	0	0	0	0	0	0	0	0	0	0
zbp	1	1	1	1	1	1	0	0	0	0	0	0	0	0	0	0
zjg	1	1	1	1	1	1	0	0	0	0	0	0	0	0	0	0
ybp	1	1	1	1	1	1	0	0	0	0	0	0	0	0	0	0
yjg	1	1	1	1	1	1	0	0	0	0	0	0	0	0	0	0
wz	1	0	0	0	0	0	1	0	0	0	0	0	0	0	0	0
FC	0	0	0	0	0	0	0	1	0	0	1	0	0	0	0	0
FS	0	0	0	0	0	0	0	0	1	0	0	0	0	1	0	0
T0	0	0	0	0	0	0	0	0	0	1	1	0	0	0	0	0
T1	0	0	0	0	0	0	0	1	0	0	1	0	0	0	0	1
T2	0	0	0	0	0	0	0	0	0	0	0	1	0	0	0	0
T30	1	0	0	0	0	0	0	0	0	0	0	0	1	0	0	0
T31	1	0	0	0	0	0	0	0	0	0	0	0	0	1	0	0
T32	0	0	0	0	0	0	0	0	0	1	0	0	0	0	1	0
T5	0	0	0	0	0	0	0	0	0	0	0	1	0	0	0	1

Note: A value of 1 indicates a directed edge from the row node to the column node; 0 indicates that no directed engineering relationship is specified.

**Table 3:** Comparative results of GasNet and baseline models.

Model	Precision	Recall	F1-Score	FAR	FNR	FP
Transformer	0.748	1	0.855	0.0042	0	35
Autoformer	0.812	0.875	0.842	0.0025	0.125	21
Crossformer	0.805	0.875	0.838	0.0026	0.125	22
DLinear	0.866	0.75	0.804	0.0014	0.25	12
FEDformer	0.819	0.875	0.846	0.0024	0.125	20
FiLM	0.812	0.75	0.774	0.0039	0.25	33
Informer	0.852	1	0.921	0.0021	0	18
GasNet	0.881	1	0.937	0.0017	0	14

**Table 4:** Performance comparison of GasNet and ablated models.

Model	Precision	Recall	F1-Score	FAR	FNR	FP
GasNet	0.8814	1	0.9361	0.0017	0	14
GCN-only	0.4375	0.875	0.5839	0.0141	0.125	117
TimesNet-only	0.619	0.375	0.4684	0.0029	0.625	24
GasNet without prior adjacency matrix	0.7143	0.625	0.6667	0.0032	0.375	26
GCN without prior adjacency matrix	0.574	0.625	0.5981	0.0058	0.375	48

## Data Availability

The data presented in this study are not publicly available due to confidentiality agreements with the cooperating coal mine. The data may contain sensitive production and safety-monitoring information and therefore cannot be shared publicly.
